# Cell Contact–Dependent Outer Membrane Exchange in Myxobacteria: Genetic Determinants and Mechanism

**DOI:** 10.1371/journal.pgen.1002626

**Published:** 2012-04-12

**Authors:** Darshankumar T. Pathak, Xueming Wei, Alex Bucuvalas, Daniel H. Haft, Dietlind L. Gerloff, Daniel Wall

**Affiliations:** 1 Department of Molecular Biology, University of Wyoming, Laramie, Wyoming, United States of America; 2 J. Craig Venter Institute, Rockville, Maryland, United States of America; 3 Department of Biomolecular Engineering, University of California Santa Cruz, Santa Cruz, California, United States of America; Uppsala University, Sweden

## Abstract

Biofilms are dense microbial communities. Although widely distributed and medically important, how biofilm cells interact with one another is poorly understood. Recently, we described a novel process whereby myxobacterial biofilm cells exchange their outer membrane (OM) lipoproteins. For the first time we report here the identification of two host proteins, TraAB, required for transfer. These proteins are predicted to localize in the cell envelope; and TraA encodes a distant PA14 lectin-like domain, a cysteine-rich tandem repeat region, and a putative C-terminal protein sorting tag named MYXO-CTERM, while TraB encodes an OmpA-like domain. Importantly, TraAB are required in donors and recipients, suggesting bidirectional transfer. By use of a lipophilic fluorescent dye, we also discovered that OM lipids are exchanged. Similar to lipoproteins, dye transfer requires TraAB function, gliding motility and a structured biofilm. Importantly, OM exchange was found to regulate swarming and development behaviors, suggesting a new role in cell–cell communication. A working model proposes TraA is a cell surface receptor that mediates cell–cell adhesion for OM fusion, in which lipoproteins/lipids are transferred by lateral diffusion. We further hypothesize that cell contact–dependent exchange helps myxobacteria to coordinate their social behaviors.

## Author Summary

All cells interact with their environment, including other cells, to elicit cellular responses. Cell–cell interactions between eukaryotic cells are widely appreciated as large multicellular organisms coordinate cell behaviors for tissue and organ functions. In bacteria cell–cell interactions are not widely appreciated, as these organisms are relatively simple and are often depicted as single-cell entities. However, over the past decade, the concept of bacteria living in microbial communities or biofilms has received broad acceptance as a major lifestyle. As biofilm cells are packed in tight physical contact, there is an opportunity for cell–cell signaling to provide spatial and physiological clues of neighboring cells to elicit cellular responses. Although much has been learned about diffusible signals through quorum sensing, little is known about cell contact–dependent signaling in bacteria. In this report we describe a new mechanism where bacterial cells within structured biofilms form contacts that allow cellular material to be exchanged. This exchange elicits phenotypic changes, including in cell movements and development. We hypothesize that OM exchange involves kin recognition that bestows social benefits to myxobacterial populations.

## Introduction

Biofilms are ubiquitous in nature. Within these structures microbes adhere to surfaces and each other in dense communities coated by an extracellular matrix. Although biofilms are of great medical and industrial interest [1], little is known about how these cells interact. In some cases, cell-cell contacts likely promote communication and provide spatial cues about neighboring cells to direct biofilm maintenance and maturation [2], [3]. Experimentally, biofilm research is hindered by limited knowledge and approaches to study their cellular dynamics [4]. Recently we described a novel biofilm dependent process whereby myxobacteria exchange their outer membrane (OM) lipoproteins [5], [6]. This transfer process can result in phenotypic changes and may represent a unique mechanism in which biofilm cells communicate. Although OM lipoprotein exchange is an interesting phenomenon, little is known about the mechanism and protein components required for transfer.

Myxobacteria are gram-negative soil dwelling microbes that exhibit complex multicellular behaviors. Central to these behaviors is gliding motility, which powers and coordinates swarm expansion, rippling, predation and fruiting body development on solid surfaces. *Myxococcus xanthus* has two distinct motility systems called A (adventurous) and S (social) motility, which served as the experimental backdrop for the discovery of OM lipoprotein exchange [7], [8]. S-motility is powered by the retraction of type IV pili adhered to external surfaces, effectively pulling the cell forward [9]. The motor powering A-motility is beginning to be defined and may involve cell surface adhesins that translocate on tracks [10]. Nonmotile mutants (A**^−^**S**^−^**) thus typically contain two mutations. Of interest here is a small subset of motility mutants that can be complemented extracellularly when mixed with another strain that encodes the corresponding wild-type gene [8], [11]. Historically, this process was called ‘stimulation’ as the recipient mutant transiently gains the ability to glide. Stimulation only involves phenotypic changes; there are no genotypic changes. Of the six stimulatable motility genes (*cglB/C/D/E/F* and *tgl*) [7], [8], only two have been previously identified; *cglB* (A-motility) and *tgl* (S-motility) [12], [13]. Importantly, both of these genes encode type II signal sequences (SS) for lipoproteins. The mechanism of stimulation was determined to involve cell-to-cell transfer of either the CglB or Tgl lipoproteins from donor to recipient cells, thus restoring missing protein function to the respective mutant [5]. Strikingly, lipoprotein transfer is efficient as recipient cells accumulate approximately equal quantities of proteins as donors [5], [6]. Recently, we described the identification of the *cglC*/*D*/*E*/*F* genes [14]. These genes encode either a type I or type II signal sequence.

To determine the molecular mechanism of OM lipoprotein exchange (stimulation) we recently defined the *cis* factor requirement in the cargo protein [6]. Surprisingly, simply a type II signal sequence for OM localization is sufficient for heterologous transfer of the mCherry fluorescent protein. Cytoplasmic or inner membrane reporters were not transferred. Transfer also requires specific cell-cell contacts where motility is apparently required to align biofilm cells [6], [15]. Here, we sought to identify *trans* or host genetic determinants required for lipoprotein transfer. In a prior study we screened known S-motility mutants for stimulation defects [16]. This resulted in the identification of a subset of *pil* mutants that were conditionally defective in *tgl* stimulation. However, these mutants were not further pursued because they are functional for *cgl* stimulation, and *tgl* stimulation occurs when cells are mixed on hard agar at low cell densities. This report identifies two gene products universally required for stimulation and lipoprotein transfer. In addition, we provide evidence, for the first time, that myxobacteria exchange their OM lipids, and that this process can regulate swarming and developmental behaviors.

## Results

### Identification of TraA, a protein universally required for stimulation and lipoprotein transfer

To elucidate the mechanism of lipoprotein transfer we sought to identify mutants defective in stimulation. We reasoned that *cgl* and *tgl* stimulation occurs by a common mechanism, whereby OM proteins, and perhaps periplasmic proteins, are transferred from donor to recipient cells that lack a corresponding protein function. To avoid trivial or idiosyncratic mutants associated with particular *cgl* or *tgl* genes, we sought mutants universally defective in stimulation of the six known *cgl*/*tgl* complementation groups. We initiated these studies by first characterizing select mutants in the Dale Kaiser strain collection, the laboratory in which A- and S-motility and stimulation were discovered [7], [8]. One such mutant (DK396), isolated by Jonathan Hodgkin, appeared to possess the desired phenotype. This strain was isolated by ultraviolet light mutagenesis on an A^−^S^+^ (DK1211) strain and then screened for the loss of S-motility (nonmotile A^−^S^−^). Serendipitously, this mutant was found to be donor defective for stimulation, a phenotype we verified for all *cgl*/*tgl* mutants.

**Figure 1:**
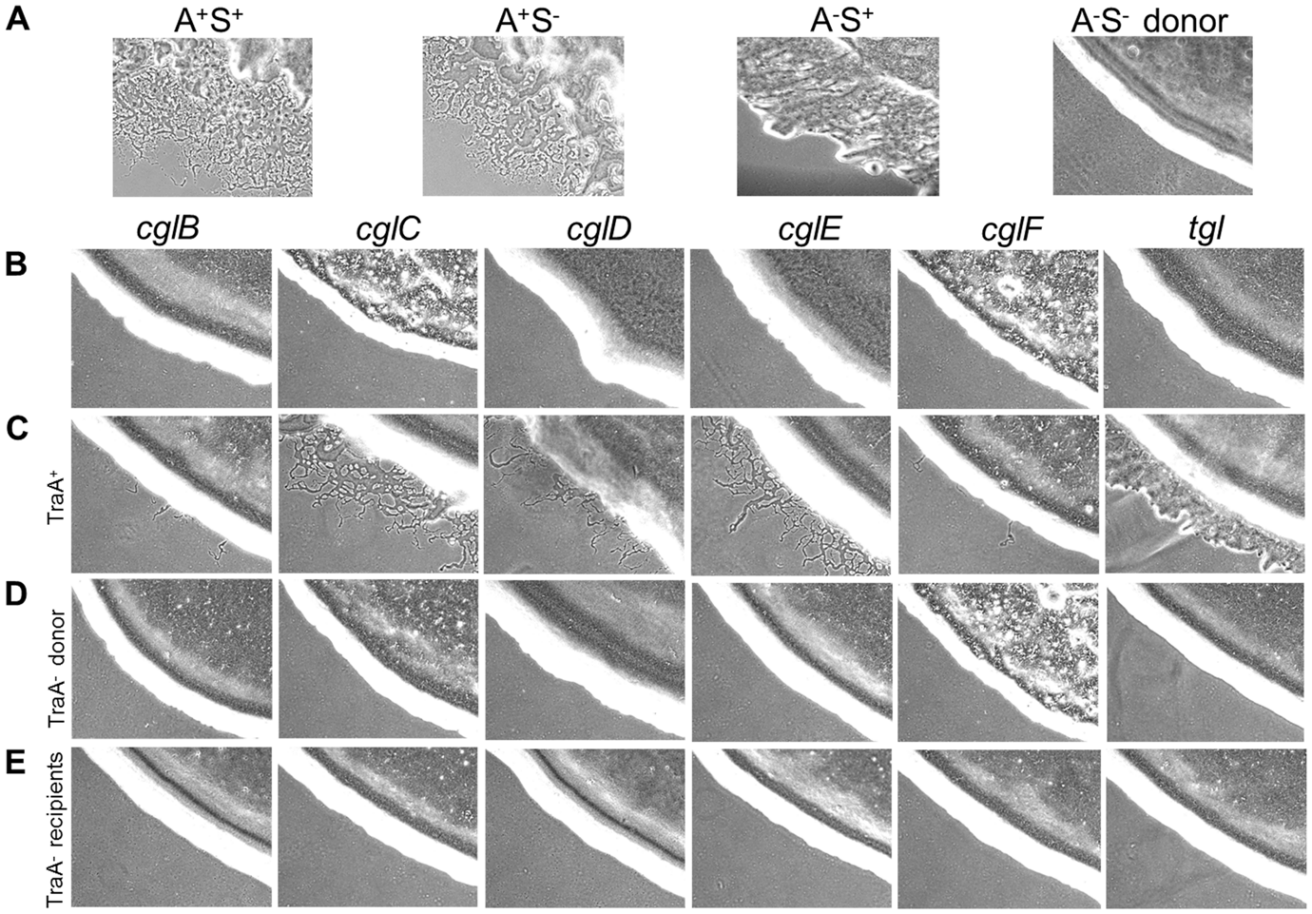
Stimulation of gliding motility depends on TraA. (A) Indicated motility phenotypes shown for reference. To easily observe stimulation the donor strain (DK6204) was nonmotile (A**^−^**S**^−^**), i.e. produces sharp colony edges, and nonstimulatable, while the (B) *cgl* and *tgl* recipients were phenotypically A**^−^**S**^−^** but stimulatable. (C) Indicated mutants mixed 1∶1 with the donor exhibiting various degrees of A- or S-motility stimulation. (D) Same as C, except strains mixed with isogenic *traA*::km donor. (E) Same as C, except isogenic recipient strains contain the *traA*::km mutation. Cells were incubated for 22 hrs at 33°C and observed with 10× objective. Table S2 lists strain genotypes.

As the donor defect mutation was not known nor easily mapped, the DK396 genome was sequenced to identify the gene of interest. Upon >39X sequence coverage the DK396 genome was compared to the wild-type DK1622 genome to identify DNA changes [17]. Mutations in 20 gene candidates were identified (Table S1). The mutations responsible for the A- and S-motility defects, but not the stimulation defect, were easily found as they were in known motility genes (Table S1; *aglT* and *pilR*, respectively) [18], [19]. Based on the severity of the mutations and predictions of gene function and subcellular localization, a prioritized list of 9 gene candidates was chosen. Assuming the phenotype was caused by a loss-of-function mutation, these genes were systematically tested for a role in stimulation by a rapid gene disruption method in a nonmotile donor strain. From these experiments one insertion mutation in *mxan_6895* (hereby named *traA* for transfer) was found to recapitulate the donor defective phenotype observed in DK396. Figure 1 shows that a disruption mutation in *traA* results in a complete block of stimulation for all the *cglB*, *C*, *D*, *E*, *F* and *tgl* mutants, as indicated by sharp colony edges (Figure 1 row D). The *traA^+^* isogenic control donor stimulates all *cgl*/*tgl* mutants for A or S-motility (Figure 1 row C). The degree to which strains were stimulatable varied and only involved partial motility restoration (Figure 1 compare row C to A). From these results it was concluded that *traA* was universally required for *cgl*/*tgl* stimulation.

**Figure 2:**
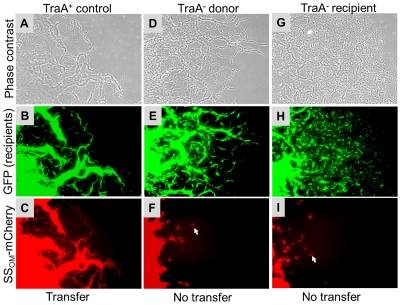
Transfer of heterologous SS_OM_-mCherry reporter in *M. xanthus* swarm requires TraA. Nonmotile SS_OM_-mCherry donors (DW1411 or DW1412) were mixed 1∶3 with A-motile GFP labeled recipients (DW1414 or DW1416). After 1 day incubation on TPM agarose pad A-motile recipients readily swarm out from the inoculum spot. Column micrographs were of identical fields taken under phase contrast and GFP or mCherry fluorescence (20× objective). Indicated isogenic strains contain *traA^+^* or *traA*::km alleles. In panels F and I arrows indicate nonmotile donor cells that were pushed or dragged to the swarm edge [6]. Table S2 lists strain genotypes.

**Figure 3:**
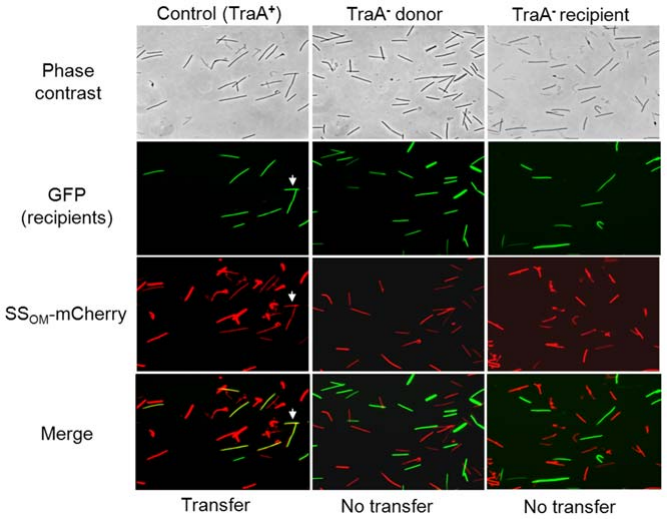
SS_OM_-mCherry transfer requires TraA. A 1∶1 mixture of donor and recipient cells were mixed and spotted on ½ CTT 1% agar and incubated for 4 hrs. Swarms were harvested and single cells were microscopically examined on glass slides to test whether GFP recipients became red by obtaining SS_OM_-mCherry. White arrows highlight two cells where transfer occurred. Column micrographs (100× objective) were of identical fields. Rod shaped *M. xanthus* cells were ∼0.5×6.0 microns. Strains were as described in Figure 2 and were *traA^+^* unless indicated otherwise in column headers.

Next we tested whether TraA was required for SS_OM_-mCherry transfer [6]. This reporter has a type II SS for OM lipoprotein localization fused to a fluorescent protein. In this assay a nonmotile and non-stimulatable SS_OM_-mCherry donor was mixed with an A-motile GFP^+^ labeled recipient. The cell mixture was pipetted onto a TPM agarose pad and motile recipients were allowed to swarm. The swarm edge was then examined by epifluorescence microscopy to determine whether SS_OM_-mCherry was transferred from the nonmotile donor to motile recipients. As shown in Figure 2C controls, SS_OM_-mCherry was readily detected in GFP labeled recipient flares [6]. In contrast, an isogenic donor that contained the *traA*::km disruption exhibited no SS_OM_-mCherry transfer (Figure 2F). To verify these results we conducted related experiments where the same strains were again mixed and spotted on agar, and after short incubations cells were harvested and microscopically examined on glass slides. Here transfer was directly tested by assessing whether GFP labeled recipients become red. As previously reported, control strains show transfer (Figure 3 left green and red panels, see arrows), where typically >90% of recipients obtain detectable levels of SS_OM_-mCherry [6]. In contrast, when an isogenic *traA^−^* donor was used no SS_OM_-mCherry transfer was detected (Figure 3 middle merged panel). We further note that replication of this experiment; under similar or different conditions/strain backgrounds, where thousands of cells were evaluated, never resulted in detection of SS_OM_-mCherry transfer from a *traA^−^* donor. We conclude that TraA is required for OM lipoprotein transfer and stimulation.

### Genetic analysis of the *traAB* operon

**Figure 4:**
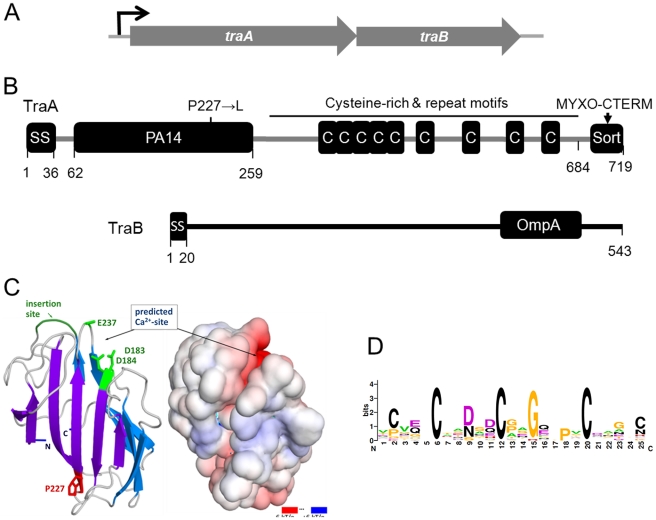
Genetic and modular structure of TraAB. (A) Operon structure depicting genes that translationally overlap. (B) Domain and motif architecture and the DK396 amino acid substitution indicated. (C) Modeled three-dimensional structure and electrostatic surface potential of TraA PA14 domain. Features shown in green in the ribbon diagram (left) could serve to recognize glycans through potential side-chain coordination of a calcium ion by Asp183, Asp184, Glu237 (only Cα-Cβ shown), and the location of an insertion important for carbohydrate-binding specificity in FLO5 [24]. Graphics produced with PyMOL (Molecular Graphics System, Version 1.3, Schrödinger, LLC) and APBS Tools2 [61]. (D) consensus sequence LOGO [62] for Cys-repeats found in TraA and myxobacteria family members designated TIGR04201.

The *traA* ORF and the downstream *mxan_6898* ORF (locus tag numbers are not consecutive) overlap by four bases, suggesting they form an operon and their gene products may function in the same pathway (Figure 4A). To test this we created an insertion mutation in *mxan_6898*. This mutant exhibited a complete block in stimulation for all *cgl* and *tgl* mutants and was completely defective in SS_OM_-mCherry transfer (Figures S1 and S2). In addition, markerless in-frame deletions in *traA* and *mxan_6898* were constructed and found to elicit the identical phenotypes reported here. Therefore *mxan_6898* was named *traB* and its gene product is predicted to function in the same pathway as TraA.

The *mxan_6894* ORF is located 126 bps upstream of *traA*, suggesting it is not part of the *traAB* operon. To test for a possible role in stimulation/transfer an insertion mutation was again created. In contrast to *traA* and *traB*, the *mxan_6894*::km mutant showed no overt defect in simulation or SS_OM_-mCherry transfer. To test whether the stimulation/transfer defect of DK396 was solely caused by the *traA* mutation, the selectable *mxan_6894*::km mutation and the tightly linked *traA^+^* allele were transduced into DK396. All resulting Km^r^ transductants were fully competent for stimulation, thus the *traA* mutation in DK396 caused the stimulation/transfer defects found in this strain. Since the mutation in DK396 was a missense substitution (Table S1; 227P→L), we tested whether it caused a dominant-negative phenotype by complementation analysis. Here, the wild-type *traAB* genes were cloned into a plasmid that directs site specific recombination into the Mx8 phage attachment site. Integration of this plasmid into the DK396 genome restored stimulation to the resulting strain, thus demonstrating the *traA227P→L* allele was recessive. In addition, this plasmid, which has *traAB* under the heterologous transcription control of the strong *pilA* promoter, was introduced into a *tra^+^* strain that contains the SS_OM_-mCherry reporter. Strikingly, upon microscopic examination this TraAB overexpressing strain was found to dramatically cause cells to adhere to one another in both kinked end-to-end chains and side-by-side contacts (Figure S3). The implication of this observation is discussed below.

### TraAB are required in recipients for stimulation and protein transfer

Next, we tested whether TraAB plays a role in recipient cells for stimulation/transfer. Since *traA* and *traB* mutants are fully motile, one or more of these mutations were introduced into all the *cgl*/*tgl* mutants. Importantly, when recipient cells contain a *traA* or *traB* mutation and mixed with a Tra*^+^* donor, no stimulation occurred (Figure 1 row E and Figure S2). We conclude that TraAB are required in both donor and recipient cells for stimulation. Next, defects in protein transfer were tested. As described above, when a nonmotile (*traA^+^*) donor was mixed with a motile *traA^−^* recipient, SS_OM_-mCherry was not transferred (Figure 2G–2I and Figure 3 right column). We conclude that TraAB are required in donor and recipients for stimulation and lipoprotein transfer.

#### Bioinformatic analysis

Sequence analysis of TraAB by Signal P 4.0 showed that both ORFs are predicated to encode type I SS, suggesting cell envelope localization (Figure 4B) [20]. BLAST searches against the non-redundant database revealed close homology to putative proteins in four other Myxococcales species: *Myxococcus fulvus*; *Stigmatella aurantiaca*; *Haliangium ochraceum*; and *Sorangium cellulosum* (see Figure S4 for locus names and sequences). Based on unambiguity of the top hit in each species and the extent of the sequence conservation (E-values≤1×10^−145^ over ≥89% of the query length, or more) these proteins are likely orthologs of TraA. No other orthologs emerged in our searches. Immediately downstream of the TraA SS, where the DK396 *traA227P→L* missense mutation resides (Figure 4), a region was identified with distant sequence homology to the PA14 domain (namesake from anthrax protective antigen 14 kDa) (E-value 7×10^−4^ to Conserved Domain cl08459 over residues 122 to 234). PA14 domains are found in a diverse set of eukaryotic and bacterial proteins with roles in carbohydrate binding and/or metabolism where their function is known [21], [22]. To ascertain whether the homology between TraA and PA14 was significant, an in depth bioinformatic and computational modeling analysis was undertaken. We first used the dual HMM approach HHpred [23] to confirm a match, which was strongest to the recently solved N-terminal domain of yeast Flocculin 5 [24] (HHpred P-value 6×10^−7^ over residues 148 to 245, default parameters). Various other structure prediction methods at the genesilico.pl metaserver site [25] concurred and produced top-ranked fold matches to PA14 with near-significant scores. We further tested the feasibility of this prediction by a template-based structural model, which we based on a manually extended alignment of TraA (and orthologs; see Materials and Methods), to a corrected Pfam seed alignment (Pfam07691; 35 sequences) derived from the automated predictions. Corrections to the Pfam alignment were mandated by resolvable discrepancies between the aligned sequences and superimposed structures of Flocculin-PA14 (PDB: 2XJP, FLO5_YEAST) and the “founder-type” anthrax toxin structures (e.g. PDB∶1ACC; PAG_BACAN). The alignment extract (Figure S4) shows these PA14 domains with TraA and its orthologs, and defines the modeled domain fragment (residues 62 to 259 in TraA; Figure 4). The model predicts a calcium binding site as one has been characterized in some PA14 domains but seems absent in others. This was indicated by conservation of two key residues (Asp183 and Asp184), the proximity of a possible third ligand (Glu237) in the neighboring carbohydrate-binding loop 2 (CBL2), and the electrostatic surface properties of our three dimensional coordinate model of the PA14^TraA^ (Figure 4C). Notably in Flocculin, the calcium ion at this exact site serves to specifically bind carbohydrates [24], [26]. By contrast the anthrax toxin PA14, for which no direct glycan binding has been demonstrated, lacks this calcium/carbohydrate-binding site. A regulatory calcium binding site in TraA was also consistent with our observations that stimulation was significantly enhanced by the addition of CaCl_2_ to agar, and blocked by the calcium chelator EGTA (Figure S5). From these analyses we conclude that TraA contains a *bona fide* PA14 domain. The proposed calcium-binding site and location are compatible with carbohydrate binding via this ion, a property that has so far only been established in eukaryotic PA14 domains. Within the PA14 family, PA14^TraA^ and orthologous fragments form a new and distinct myxobacterial clade.

**Figure 5:**
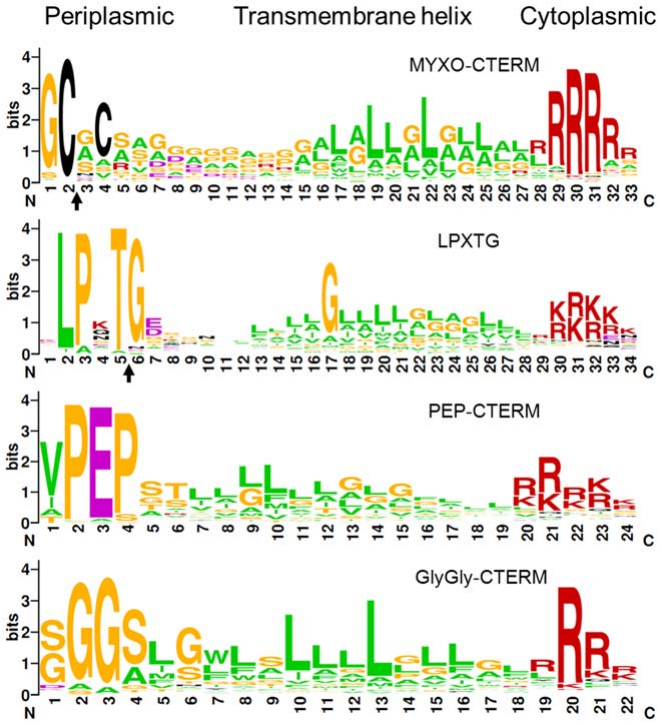
Consensus sequence LOGO of indicated bacterial C-terminal protein sorting motifs. Subcellular membrane topology predictions are shown. Black arrows indicate predicted or known proteolytic processing sites for MYXO-CTERM (TIGR03901) and LPXTG [28], respectively. Note the N-terminal conserved sequences vary between motifs, while C-terminal sequences are all enriched for arginine and lysine residues.

Following the TraA PA14 domain was a region rich in cysteines (71 Cys). Sequence analysis revealed this region contained nine repeat elements, in which the first five are in tandem (Figure 4B). Since other myxobacteria ORFs were specifically found to contain similar repeats, a new TIGRFAM was created and named TIGR04201 (Figure S6). From this a weblogo was generated to illustrate sequence diversity at each position, revealing that three Cys are invariant and two are marginally conserved (Figure 4D). As TraA was predicted to be secreted, these cysteines are likely oxidized to form disulphide bonds. It is also noteworthy that cysteine-rich proteins are characteristically associated with the extracellular matrix [27]. Lastly, we discovered that TraA encodes a myxobacteria-specific C-terminal motif (Figure 4B). Within the DK1622 genome we identified 34 ORFs (Figure S7) that contained this motif we called MYXO-CTERM and it was designated as a new TIGRFAM named TIGR03901. These sequences were again aligned to generate a weblogo (Figure 5). This MYXO-CTERM motif contains a predicted transmembrane α-helix and as such the C-terminal residues, rich in Arg, are likely cytoplasmic. In contrast, the N-terminal residues are likely periplasmic, including position 2 which contains an invariant Cys (Figure 5 and Figure S7). Comparative logos were created to other bacterial C-terminal tags known or postulated to function in protein sorting. As graphically depicted these logos show MYXO-CTERM shares striking sequence and membrane topology similarities (Figure 5). The best studied example is LPXTG (TIGR01167), a protein sorting tag widely found in gram-positive bacteria that results in processing by sortase and subsequent covalent attachment to the cell surface [28]. Similarly, the PEP-CTERM (TIGR02595) and GlyGly-CTERM (TIGR03501) motifs are predicted to be involved in protein sorting and cell surface localization in gram-negative bacteria [29]–[31]. Thus, by analogy to the LPXTG, PEP-CTERM and GlyGly-CTERM tags, the MYXO_CTERM was postulated to serve as a myxobacteria specific protein sorting tag for cell surface localization. Consistent with this, 9 of the 34 *M. xanthus* TIGR03901 ORFs were experimentally found on the cell surface [32]. Separately, the N-terminal region of TraB showed no significant homologies, while its C-terminal region encodes an OmpA/MotB-like domain (Pfam00691), presumably involved in peptidoglycan binding.

As described above *traA* encodes a protein with unique bacterial domain architecture, yet surprisingly *mxan_4924* encodes a close paralog (BLAST E-value 4×10^−101^ against NCBI non-redundant database, over TraA residues 40 to 364) with very similar domain architecture; type I SS, PA14 domain, cysteine-rich repeats (TIGR04201) and MYXO-CTERM. Because of these similarities an insertion mutation was created, *mxan_4924*::km. However this mutant exhibited no overt defect in stimulation or SS_OM_-mCherry transfer and consequently has no ascribed function.

### TraAB are required for OM lipid exchange

**Figure 6:**
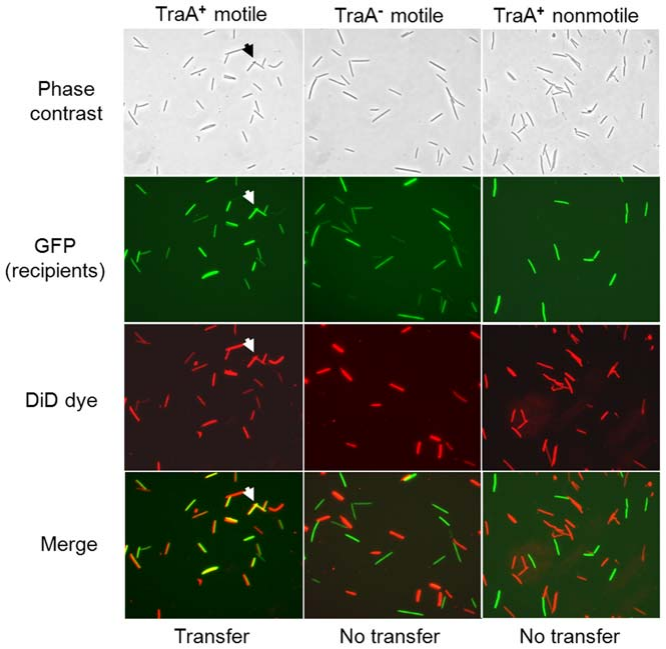
Lipophilic fluorescent dye (DiD) transfer depends on TraA and cell motility. Column headers list relevant properties of isogenic recipients. The same nonmotile (DK8601) donor was used in all mixtures and recipients were DW1414 (A^+^ *traA^+^*), DW1416 (A^+^ *traA^−^*) and DK8606 (A^−^ *traA^+^*). The ability of the DiD dye (red fluorescence) to be transferred to GFP recipients was assessed in merged panels (100× objective). Here yellow color indicates DiD transfer to GFP recipients and representative cells are noted by arrows.

The finding that OM lipoproteins are efficiently and apparently non-specifically transferred suggests that OM lipids may also be exchanged. To test this, donor cells were stained with a fluorescent lipophilic dye called DiD oil. As shown, DiD specifically stained the cell envelope, which fluoresced red (Figure S8). Importantly, when stained cells were harvested, washed and mixed with GFP labeled recipients in solution, recipients did not fluoresce red, indicating the dye did not freely diffuse between cells. As transfer requires a hard surface, cell-cell contact and motility, we next tested, under these conditions, for DiD transfer [6]. As shown in Figure 6 (left panels), DiD transfer readily occurred to GFP-labeled recipients. As controls, no DiD transfer occurred when isogenic recipients contained a *traA* mutation or when donor and recipients were both nonmotile (Figure 6, middle and right panels, respectively). In accordance with the above results, TraA was also required in donors, and similarly TraB in donors/recipients, for DiD transfer (Figure S9). These experiments show, similar to SS_OM_-mCherry transfer (Figure 2 and Figure 3) [6], that lipophilic dye and hence OM lipid, requires a hard surface, cell motility, and TraAB functions in donor and recipient cells for transfer.

### OM exchange can regulate motility and development behaviors

**Figure 7:**
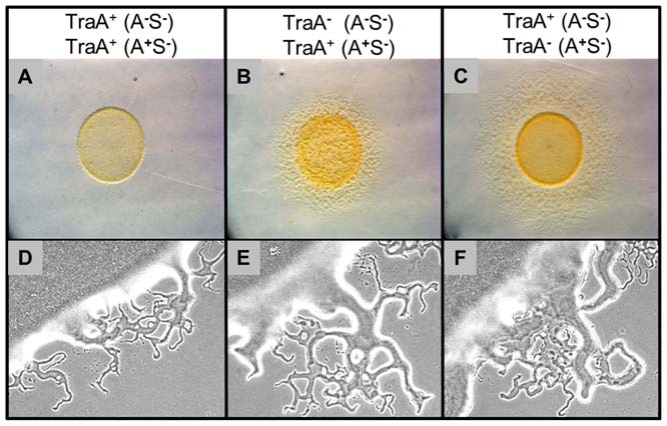
Outer membrane exchange regulates mixed colony swarm expansion. Column headers list relevant strain features of nonmotile DK8601 (A, C, D and F) or DW1419 (B and E) and A-motile DK8615 (A, B, D and E) or DW1415 (C and D) isogenic strain mixtures (1∶1 ratios). Top panels are 4 day old colonies (stereo micrographs). Bottom panels are colony edges of identical strain sets at higher magnification (10× phase contrast objective) taken at an earlier time point (15 hrs). Swarm expansion of the A^+^ strain was inhibited by a nonmotile strain in a TraA dependent manner.

As noted above, the *traA* and *traB* mutants exhibited no overt defects in A or S-motility, suggesting that OM transfer was not required for motor functions. However, the exchange of OM lipids and proteins involves significant resource sharing between cells and therefore this process must involve physiological consequences. One such phenotypic consequence was the restoration of swarming defects to certain motility mutants (Figure 1). However, extracellular complementation might have little significance between wild-type cells as they contain a full complement of motility proteins. In strain-mixing experiments we discovered that *tra* ***^+^***, but not *tra* ***^−^*** strains, dramatically inhibited swarm expansion when a nonmotile strain was mixed with a motile strain. An example of how a nonmotile strain inhibits swarm expansion of an A^+^S^−^ strain was illustrated in Figure 7A. In contrast, when identical mixing experiments were done between isogenic *traA^−^* strains, swarm expansion occurred (Figure 7B and 7C). As was found for lipoprotein and lipid transfer, the relief of swarm inhibition occurred when the *traA* mutation was introduced into either the nonmotile or motile strains. However, we note, swarm expansion was consistently more robust when the motile strain, instead of the nonmotile strain, contained the *traA* mutation (compare Figure 7B to 7C). An identical relief of swarm inhibition was again found when strains instead contained the *traB* mutation. Similarly, a Tra^+^ dependence for swarm inhibition of A^+^S^+^ motility was found when these strains were instead mixed with a nonmotile strain. In contrast, inhibition of A^−^S^+^ motility was minimal. To test whether swarm inhibition was specific to certain motility genes we test a variety of A**^−^**S**^−^** double mutants, including combinations of *dsp/dif*, *pilA*, *pilM*, *pilT*, *pilQ*, *stk*, *aglB*, *aglR* and *aglM* mutations, and in all cases these nonmotile strains inhibited swarm expansion of A^+^S^−^ motile strains. We conclude that swarm inhibition was not dependent on specific motility genes, but instead was dependent on TraAB and thus OM exchange.

Macroscopically swarm inhibition was apparent (Figure 7A, 4 day incubation); however swarm inhibition was not absolute as flares were initially observed emerging from inoculation mixtures (Figure 7D, 15 hrs). Microscopically, the number and size of these early emerging flares were reduced compared to *traA^−^* mixtures (Figure 7, compare 7D to 7E and 7F). However, over longer incubations, e.g. 4 days, the strain mixtures that were Tra^+^ failed to swarm farther (Figure 7, compare 7A to 7B and 7C). To investigate this behavior time-lapse microscopy was used to track cell movements. Consistent with the above observations, for the first ≥1 day after plating the A-motile cells exhibited similar cell movements with respect to speed, reversal frequency and percent of cells moving, whether the mixtures contained *tra^+^* or *tra^−^* cells. In contrast, by day 2 these same cell mixtures exhibited drastically different behaviors. That is mixtures containing *traA^+^* cells exhibited a complete block in group movements, while isolated cells occasionally exhibited motility that was aberrant (Video S1). In sharp contrast, isogenic strain mixtures with *traA^−^* mutations in either the motile or nonmotile strain exhibited robust group and single cell motility (Videos S2 and S3). Swarm inhibition does not appear to depend on a diffusible signal, because when these identical *tra^+^* strains were separated by a membrane (nitrocellulose) or soft agar overlay, no motility inhibition was observed. Hence, we hypothesize that nonmotile cells produce a time dependent (≥2 days) physiological signal that was transferred by OM exchange to motile cells that blocked their motility.

**Figure 8:**
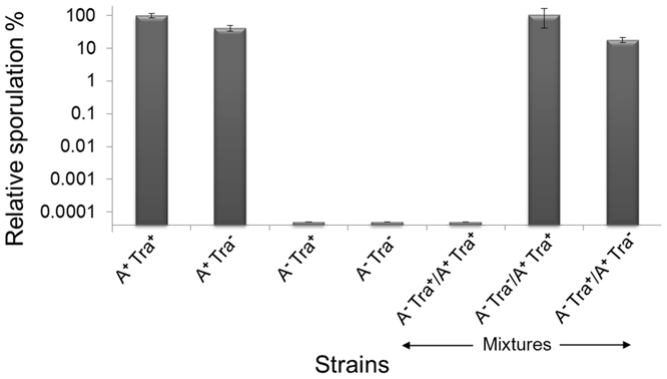
Outer membrane exchange regulates developmental sporulation. An A^+^S^−^ strain (DK8615) designated A^+^ Tra^+^ was sporulation proficient and arbitrarily set at a 100%; while a nonmotile strain (A^−^ Tra^+^; DK8601) did not sporulate. The *traA*::km mutation (Tra^−^) was crossed into these strains (DW1415 and DW1419, respectively) and their sporulation efficiencies did not significantly change. A-motile and nonmotile strains were then mixed at a 1∶1 ratio and sporulation of the A^+^ strain was blocked in a Tra^+^ dependent manner. Development was conducted on TPM starvation agar and cells were harvested after 5 days and viable spores determined in triplicate, averaged and error bars shown.

Myxobacteria are noted for the social behaviors and ability to form multicellular fruiting bodies in response to starvation. We thus tested whether Tra plays a role in development. A *traA* mutation was introduced into a wild-type strain, but no overt defects in fruiting body formation or sporulation was observed. To extend the above swarm inhibition findings, we next tested whether genetically distinct strain mixtures, as found in nature [33], interfered with development in a Tra dependent manner. First, the *traA* mutation did not significantly alter the ability of A^+^S^−^ strain to sporulate (Figure 8) [34]. Second, as development is coupled to motility [35], nonmotile strains cannot fruit or sporulate and a *traA* mutation does not alter this phenotype (Figure 8). Strikingly, however, when the A^+^S^−^ strain was mixed in a 1∶1 ratio with a nonmotile strain no viable spores were detected (≥6-logs; Figure 8). In contrast, when isogenic strains contained the *traA* mutation in either strain, the ability of the A-motile strain to sporulate was restored to control levels (Figure 8). Thus similar to swarm inhibition, a nonmotile strain can block development of a motile strain that depends on TraA and hence OM exchange.

## Discussion

To understand the mechanism of lipoprotein exchange we identified mutants universally defective in *cgl*/*tgl* stimulation and protein transfer. Interestingly, these TraAB proteins were required in both donor and recipient cells. To our knowledge, this is the first bacterial transfer system where the same gene products are required in both donor and recipient cells. This finding and the ability of *M. xanthus* cells to rapidly and homogeneously exchange lipoproteins [5], [6] implies that lipoproteins are transferred in a bidirectional manner. A bidirectional transfer mechanism is distinct from known secretion and conjugative systems [36], [37], where proteins or DNA are transferred unidirectionally from donor to recipient cells.

Since OM lipoprotein exchange occurs efficiently and involves a form of bulk transfer [5], [6], we hypothesized that OM lipids may also be exchanged. This hypothesis was supported by the finding that a lipophilic fluorescent dye was readily exchanged between cells. Importantly, transfer of lipophilic dye and hence membrane lipids, have the same stringent requirements in transfer as OM lipoproteins [6]. That is, dye transfer only occurred when cells were motile within structured biofilms; no detectable dye transfer occurred in liquid or between nonmotile (non-stimulatable) cells on a solid surface. In addition, dye transfer required the TraAB proteins in donor and recipient cells. We thus conclude that dye exchange does not occur by diffusion or by diffusible OM vesicles, but instead requires specific cell-cell contacts mediated by cell motility. Based on earlier observations that OM, but not IM, lipoproteins are transferred [6], we surmise that only OM lipids are exchanged bidirectionally. Presumably transfer consists of the outer leaflet lipopolysaccharide (LPS) and the inner leaflet phospholipids. In this respect it is interesting to note that species of *Borrelia* have been directly observed to fuse their OMs, a process apparently mediated by cell motility [38], and *Bacillus subtilis* reportedly transfers proteins in biofilms via membrane enclosed nanotubes [39].

Based on sequence, domain architecture and functional similarities to eukaryotic proteins, we propose that TraA serves as a cell surface receptor. In particular, TraA has similarities to the *Saccharomyces cerevisiae* FLO1 and FLO5 cell surface receptors/adhesions [21], [24], [40] (Figure 4). These FLO proteins have domain architecture consisting of a SS, N-terminal PA14 domain, a central tandem repeat region and a C-terminal protein sorting tag (GPI site; glycosylphosphatidylinositol anchor) for cell surface attachment [26]. Thus, by analogy, we suggest that in TraA the SS serves to transport the protein to the periplasm followed by SS cleavage. The processed N-terminal PA14 domain would serve as a receptor for ligand binding, presumably a glycan. The cysteine-rich tandem repeats could serve as a rigid stalk for PA14 presentation on the cell surface. The MYXO-CTERM motif could function, analogous to a GPI site, in protein sorting to the cell surface. Recent reports suggest the MYXO-CTERM and related C-terminal tags (Figure 5) are widely distributed in bacteria and archaea, where they are proposed to be posttranslationally modified and direct protein sorting to the cell surface [29]–[31]. Although initial attempts to generate TraA antibodies or fluorescent protein fusions were unsuccessful, TraAB overexpression was found to dramatically increase the ability of cells to adhere to one another (Figure S3). This result is consistent with TraA serving as a cell surface adhesin. Furthermore, the identification of the *traA227P→L* missense mutation within PA14 highlights the importance of this domain for function (Figure 4). We also note that *Dictyostelium discoideum*, a eukaryotic soil slime mold that exhibits similar multicellular behaviors as *M. xanthus* [41], produces two secreted signals, called DicA1 (PsiF) and PsiA, whose proteins contain PA14 domains followed by cysteine-rich repeats (Pfam00526) of various lengths that show some resemblance to TIGR04201 [21], [27], [42], [43]. Thus, *M. xanthus* and other microbes, including eukaryotes, appear to utilize PA14 encoding proteins as extracellular signaling and recognition molecules to mediate social interactions.

Recent bioinformatic analysis suggests gram-negative bacteria encode C-terminal protein sorting tags that function analogously to the well-characterized gram-positive LPXTG/sortase system [29]. In the case of MYXO-CTERM, we postulate that this motif forms a transmembrane α-helix and anchors pre-TraA into the IM [29], [31]. Here the Arg rich C-terminal tail would reside in the cytoplasm, while the remainder of the protein would be in the membrane or periplasm (Figure 5). Thus analogous to lipoprotein processing [44], an acyl transferase could attach a lipid moiety via a thioether bond to the invariant Cys (Figure 5 and Figure S7). Subsequently, an endoprotease would cleave the TIGR03901 motif downstream of the aforementioned Cys residue. Once processed a system analogous to the Lol pathway could transport these proteins to the cell surface.

As the *traB* gene overlaps in a bicistronic operon with *traA* (Figure 4A) and mutations in each gene elicit identical phenotypes, suggests that TraAB likely function in the same transport pathway. Since the C-terminal region of TraB contains an OmpA-like domain (Pfam00691), it likely binds non-covalently to the cell wall. The N-terminal region constitutes the majority of this protein (∼400 amino acids) and has no ascribed function (Figure 4), but theoretically could interact with the OM and even traverse the OM to interact with TraA. It is also plausible that TraB may facilitate TraA's localization to the cell surface.

**Figure 9:**
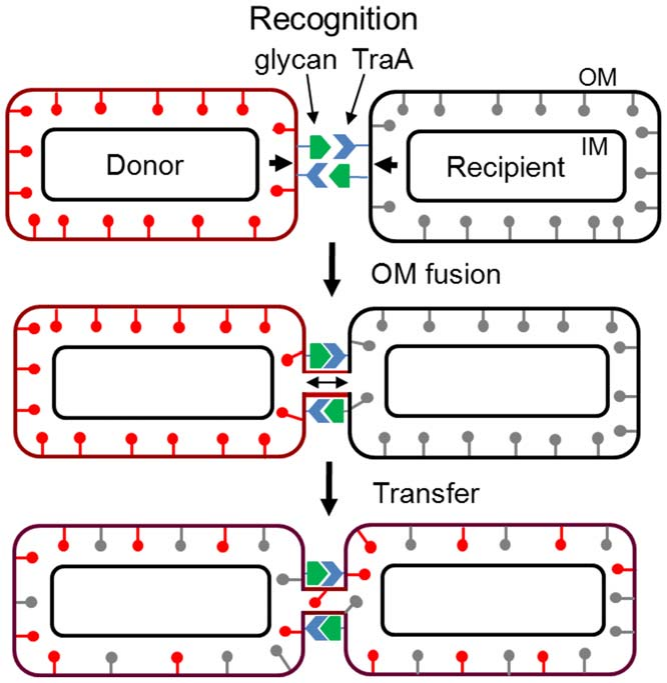
Working model for OM and lipoprotein transfer between *M. xanthus* biofilm cells. See text for details.

A working model for the mechanism of cell contact-dependent exchange is outlined in Figure 9. First, cell-cell recognition is postulated to be mediated by TraA serving as a cell surface receptor. We suggest that the distant PA14 domain may function in ligand binding to neighboring cell surfaces. Glycans found in LPS or glycoproteins are possible ligands. In a variation of this model TraA may function as a homophilic receptor. Similar to the FLO1 system, a key component of this model involves reciprocal TraA binding by both cells. A ‘donor’ cell was arbitrarily assigned and its OM (mCherry) lipoproteins were symbolized as red lollipops. Upon aligned cell-cell contact and docking the OM membranes of adjoining cells fuse. Although not directly depicted, TraAB may facilitate membrane fusion by bringing OMs into close proximity and perhaps causing local membrane perturbations that help catalyze OM fusion. Membrane fusion may also be facilitated at cell poles where the membranes have high tip curvatures and thus are more fusogenic [45]. Once cells are adhered cell motility could also stress the membrane. Upon OM fusion, lipids and lipoproteins rapidly exchange bidirectionally; a process presumably driven by lateral diffusion. Integral and associated OM proteins are also likely transferred as the CglE and CglF proteins encode type I signal sequences [14]. It is unknown whether soluble periplasmic proteins are transferred. Prior studies clearly indicate inner membrane lipoproteins and cytoplasmic proteins are not transferred [6]. Following fusion cells physically separate, a process likely facilitated by gliding motility.

The exchange of OM lipoproteins has phenotypic consequences to the cell, including complementation of mutational defects (Figure 1). Whether the restoration of mutation defects is ecologically important is unknown; however population heterogeneity within biofilms, especially from an environmental setting are significant [4], and consequently some individuals within a population are less fit. Thus, we hypothesize that the ability to exchange and share the OM proteome allows some individuals to gain fitness and for the population to establish OM homeostasis. In turn, homeostasis may increase population fitness by normalizing intercellular signal output and reception by reducing population heterogeneity. Thus community behaviors, such as swarming and development might be better coordinated. In this respect, our findings that a mixture of nonmotile cells with motile cells inhibits the latter cells from swarming in a TraAB and time dependent manner (Figure 7), suggests these cells are communicating and coordinating their behaviors via OM exchange. Similarly, OM exchange can regulate development behaviors between genetically distinct strains (Figure 8). The use of strain mixtures to study cell-cell interactions in motility and development is ecologically relevant, as diverse *M. xanthus* isolates are found in close proximity in nature [33], [46]. The mechanism for developmental inhibition by nonmotile cells on motile cells is unknown, but may simply reflect a block in motility (Figure 7) [47]. Alternatively or in addition, OM exchange with nonmotile cells may transmit a signal that blocks development. Currently, we are investigating the nature of these putative signals.

Our results indicate that myxobacteria exchange and thus share a significant amount of their cellular resources. This has led us to hypothesize that cell contact-dependent OM exchange represents a form of cooperative social behavior that may involve kin recognition. A kin recognition mechanism avoids the theoretical and ecologically relevant concern that ‘cheater’ cells could exploit or disrupt this social behavior to obtain resources [48]. This problem is highlighted by observations that environmental *M. xanthus* populations arise from diverse origins [33], [46]. Thus unlike artificial laboratory settings where multicellular behaviors are typically studied with a single homogenous culture, natural myxobacteria isolates must recognize kin from non-kin cells as they vacillate between single cell and multicellular life.

The data presented here provide three lines of evidence that cell contact-dependent OM exchange involves kin recognition. First, TraAB proteins are required in both ‘donors’ and ‘recipients.’ Thus if one cell does not express TraAB, exchange cannot occur. Second, exchange appears bidirectional, thus both cells are giving and receiving. Therefore, there is no inherent advantage one cell type has over another, unless one cell is starving and has depleted resources. Third, TraA contains a PA14 domain, with features resembling PA14 domains in yeast flocculin proteins involved in kin recognition and social behaviors. More specifically, *flo1* and other genes within this group were classified as ‘greenbeard’ genes, which by molecular definition are cell surface receptors that recognize other cells carrying the same gene to provide social preferential treatment [49], [50]. In the case of FLO1 the protein allows yeast cells to enter the protective domain of a floc, where cells are so tightly joined they become deformed. Within flocs cells are protected from environmental stresses and cheater cells (*flo1^−^*) cannot enter [40]. In another greenbeard example, the *Dictyostelium csA* gene, which encodes a homophilic cell surface receptor, plays a discrimination role in partitioning cells to desirable locations within fruiting bodies [51]. Current experiments are testing whether TraA plays such a role.

## Materials and Methods

### Strains and media

Bacterial strains and plasmids are listed in Table S2 [52]. *M. xanthus* was grown at 33°C in CTT medium (1% casitone, 1 mM KH_2_PO_4_, 8 mM MgSO_4_, 10 mM Tris-HCl, pH 7.6) in the dark and when necessary supplemented with kanamycin (Km; 50 µg/ml), oxytetracycline (Tc; 15 µg/ml), or streptomycin (Sm; 600 µg/ml). For ½ CTT, casitone was reduced to 0.5%. On plates, agar concentration was 1.0 or 1.2%. TPM buffer contains 10 mM Tris, 1 mM KH_2_PO_4_ and 8 mM MgSO_4_, pH 7.6. *Escherichia coli* was grown at 37°C in LB medium and when necessary supplemented with Km (50 µg/ml), ampicillin (100 µg/ml) or Sm (100 µg/ml).

### Genomic sequencing and genetic mapping

The DK396 genome was sequenced by using Illumina second generation DNA sequencing technology (NCGR, Santa Fe, NM). Sequence reads were aligned and analyzed for mutations against the wild-type DK1622 reference genome within the Alpheus bioinformatic platform [53].

### Genetic manipulations

DNA cloning followed routine protocols [54]. Chromosomal and plasmid DNA was isolated with UltraClean Microbial DNA and Mini Plasmid isolation kits (MO BIO Laboratories, Inc.), respectively, as described by the manufacture. All insertion mutations were created by PCR amplification of internal gene fragments with Taq 2X Master Mix (New England BioLabs) followed by direct cloning of products into pCR2.1 TOPO (Invitrogen) and then transformed into DH5α. To overexpress the *traAB* operon it was fused downstream of the strong *pilA* promoter with an optimally designed ribosomal binding site [55]. Specifically, the *pilA* promoter was amplified with Phusion High-Fidelity PCR Master Mix with HF Buffer (New England Biolabs) and cloned into pSWU19 at the *Eco*RI to *Xba*I restriction sites [18]. *traAB* was then similarly amplified and cloned into the *Xba*I and *Hind*III sites. Primers are listed in Table S3. Plasmid constructs were confirmed by restriction digestion analysis or DNA sequencing. Verified plasmids were electroporated into *M. xanthus* and integrated into the genome by homologous recombination with antibiotic selection [34]. To identify the donor defect mutation from DK396, insertion mutations were made in DK6204 [56] or DK8601 A^−^S^−^ donor strains (Table S1). Mx4 or Mx8 bacteriophages were used for strain construction by generalized transduction [15]. Mutants were verified by phenotypes and molecular methods including PCR and sequencing.

### Motility and stimulation

*M. xanthus* strains were grown to a Klett ∼100 (∼3×10^8^ cfu ml^−1^), concentrated by centrifugation and resuspended to a calculate Klett of 1000 in TPM buffer. For stimulation, donors and recipients were mixed at a 1∶1 ratio and 3 µl were pipetted onto ½ CTT 1% agar pads containing 3 mM CaCl_2_ (added after autoclaving) and incubated in a humid chamber for various times. Micrographs were taken with either an Olympus SZX10 stereo microscope (whole colony) or a Nikon E800 phase contrast/fluorescent microscope (colony edge) coupled to digital imaging systems.

### Lipoprotein transfer

A heterologous fluorescent OM lipoprotein reporter, called SS_OM_-mCherry, was used to monitor protein transfer in live cells [6]. To clearly differentiate recipients from SS_OM_-mCherry expressing donors, the former cells expressed the green fluorescent protein (GFP). Thus, in general terms, protein transfer was scored as the ability of green cells to become red. Lipoprotein transfer was microscopically determined by mixing donor and recipients (1∶3 or 1∶1 ratios) and either (i) detected as motile recipient flares emerging from inoculum spots with nonmotile donors, or by (ii) harvesting cell mixtures and inspecting single cells on glass slides as previously described [6]. To reduce background fluorescence, the former cells were spotted on a thin TPM agarose (1%) pads prepared on a glass slide.

### Fluorescent membrane staining and transfer

A sampler kit (Invitrogen; cat# L7781) containing different lipophilic fluorescent dyes were evaluated for *M. xanthus* OM staining. According to the manufacture these dyes are not transferred from stained to unstained cells. DiD oil (component B; DilC_18_(5) oil) was chosen for further studies where a Texas Red-4040B (Semrock) filter set was used to visualize staining. Cells were grown to Klett ∼100, harvested by centrifugation and resuspended in TPM buffer to a calculated Klett of 250. To stain cells, 1 µl of dye (1 mg/ml, dissolved in ethanol) was added to 49 µl of cells and incubated for 1 to 2 hrs in the dark at 33°C. Cells were then pelleted by centrifugation, washed with 1 ml TPM and microscopically examined (100× objective). Similar to monitoring SS_OM_-mCherry transfer, dye transfer was also assayed by mixing stained donors with GFP labeled recipients (1∶1 ratio) and spotted on a ½ CTT 1% agar. After 4 hrs incubation, cells were scraped from the agar surface, washed 2× in 1 ml TPM, placed on glass slide with cover-slip and inspected whether green cells also stained red.

### Development

Log phase *M. xanthus* cultures were concentrated by centrifugation to a calculated Klett of 1000 and pipetted onto TPM starvation agar (four 25 µl spots) and incubated for 5 days at 33°C. Cells and spores were harvested and placed into a tube with 500 µl of TPM buffer, heated at 50°C for 2 hrs and then gently pulse sonicated to disperse spores. Spore suspensions were serial diluted and 10 µl samples spotted on CTT agar. After 7 days of incubation, viable spores were enumerated as CFUs. All developmental assays were done in triplicate and averaged.

### TraA bioinformatics

PA14 domain analysis and alignments are described in Results and Figure S4. The cysteine-rich repeat of MXAN_6895 was identified by inspection. TIGRFAMs model TIGR04201 was developed by multiple sequence alignment of several repeats, HMM construction, search against a large collection of proteins from prokaryotic reference genomes, and iteratively refined. In proteins identified by TIGR04201 as having at least one copy of the repeat, additional, lower-scoring repeats are confirmed by manual inspection of HMM search results. Completed HMMs were added to the TIGRFAMs database, which uses the HMMER 3.0 software package [57].

A search was undertaken for candidate protein-sorting domains with architectural elements similar to the LPXTG-containing recognition sequence of sortase A [28], the PEP-CTERM putative recognition sequence of exosortase and the PGF-CTERM putative recognition sequence of archaeosortase A [29]. The common architecture was; signature motif, hydrophobic predicted transmembrane helix, cluster of basic residues, positioned at the extreme C-terminus and found in protein regions lacking other homologies. A general purpose classifier, TIGRFAMs [58] HMM TIGR03901, was constructed to model a candidate protein-sorting signal domain approximately thirty-three residues long, with an invariant Cys residue in its signature motif, universal in but restricted to the eight species of Myxococcales among 1460 prokaryotic reference genomes; scoring thresholds give no false-positive in any species. To identify atypically low-scoring instances of the domain in *M. xanthus*, a species-specific HMM was derived from TIGR03901 by HMM search, inspection of results, realignment, and repetition of the search through several iterations. Extensive biocuration of the similar but shorter GlyGly-CTERM motif found primarily in gammaproteobacteria, modeled by HMM TIGR03501, improved the disambiguation of GlyGly-CTERM (which does not occur in *M. xanthus*) from MYXO-CTERM.

### Three-dimensional structural modeling

Approximate atomic coordinates for the PA14^Tra^ structure was automatically generated from the alignment of known PA14 domains (Figure S4, residues 62 to 259). This was done by using a standard two-step template-based modeling protocol. The initial 3-D model was obtained using MODELLER 9.9, with 2XJP (FLO5) as template structure (default parameters, best-scoring of 20 models) [59]. To produce the final model, side-chain atoms were refined using SCWRL4 [60].

## Supporting Information

Figure S1 SS_OM_-mCherry transfer requires TraB. Strains used were DW1414 (GFP^+^ recipient) and DW1464 (*traB*::km SS_OM_-mCherry donor). See Figure 3 for experimental details and controls.
(TIF)

Figure S2 Stimulation of gliding motility depends on TraB. The nonmotile donor strain was DK8601. TraB^+^ recipients were DK392 (*cglD1 pilQ1*) and DK360 (*cglE1 pilQ1*). Isogenic TraB^−^ recipients were DW1465 (DK392 *traB*::km) and DW1408 (DK360 *traB*::km). Experimental conditions were as described in Figure 1.
(TIF)

Figure S3 TraAB overexpression causes cells to adhere to one another. Top panels are the parental strain DW1411, which contains a Δ*pilA* mutation that allows disperse liquid growth and the SS_OM_-mCherry reporter for fluorescent OM visualization. DW1463 is an isogenic derivative that contains a second genomic copy of *traAB* under heterologous P*_pilA_* transcriptional control (*P_pilA_*-RBS_syn_-*traAB*). Micrographs (100× objective) show identical phase contrast and fluorescent fields.
(TIF)

Figure S4 Multiple alignment extract showing Myxococcales PA14^Tra^ domain sequences with the Pfam07691 family. The *M. fulvus* ORF was manually assembled from NCBI sequences. Proteins are specified by locus tags and residue numbers. The top and bottom rows shown by their Uniprot accession codes the members with high-resolution crystal structures in the PDB: FLO5_YEAST (PDB: 2XJP), PAG_BACAN (PDB: 1ACC) specifying known β-strand locations (black arrows; strands ≥3 residues). Residue coloring follows ClustalX schema to emphasize amino acid property conservation between distant homologs. Predicted β-strands (gray arrows) are from Quick2D consensus secondary structure prediction [23] on the subfamily alignment (agreement of ≥3 methods; strands ≥3 residues). No helical segments ≥5 residues are known or predicted. Similarity to previously known PA14 domains (which was used to produce the alignment, see Results) is depicted by a LOGO [62] representation of Pfam07691 after N-terminal correction based on the known structures (seed alignment, 35 sequences; this includes the structure representatives but not the new subfamily introduced by our findings). Positions with >50% gaps were excised from the LOGO (indicated by small vertical double-lines if within a block) to avoid inadequate representation by the program. The carbohydrate-binding (CB) features characterized in the flocculins [22], [24] are annotated above FLO5 (dark red triangles and lettering; CBL1 and CBL2, carbohydrate-binding loops with directly Ca^2+^ coordinating side chains). Red unfilled symbols markup features in the new PA14^Tra^ subfamily that could serve a similar purpose based on homology and/or structural analogy rationales.
(TIF)

Figure S5 Calcium chloride enhances stimulation. A nonmotile non-stimulatable donor strain (DK8601) was mixed with the respective nonmotile but stimulatable recipient strains; DW1466 (Δ*cglC* Δ*tgl*::tc), DK8602 (*aglB1* Δ*tgl*::tc) and DK1633 (*cglC1 pilQ1633*). Note, DW1466 can be stimulated for both A- and S-motility. Top panel assays were conducted on ½ CTT 1.0% agar, while the middle and bottom panels contained the same media with indicated supplements. See Figure 1 for details.
(TIF)

Figure S6 Alignment of Myxococcales Cys-rich repeats (TIGR04201). Locus tag and amino acid positions are given. Top nine sequences are from TraA (MXAN_6895), in which the first five are tandem repeats. Light blue highlights indicates highly conserved Cys residues, while dark blue highlights indicates less conserved Cys residues. Grey box shading highlights other conserved residues.
(TIF)

Figure S7 Alignment of MYXO-CTERM motif (TIGR03901) from *M. xanthus* DK1622 genome. Locus tags and residue positions are shown. Conserved residues highlighted.
(TIF)

Figure S8 Lipophilic DiD dye stains the outer membrane. Cells observed with 100× objective.
(TIF)

Figure S9 Lipophilic fluorescent dye (DiD) transfer depends on TraB in donor and recipient cells. Strains are *traB^+^* unless indicate otherwise. The *traB^−^* strain was DW1417. See Figure 6 for details.
(TIF)

Table S1 Mutations identified in DK396 genome.
(XLSX)

Table S2 Plasmids and strains used in this study.
(DOCX)

Table S3 Primers used in this study.
(DOCX)

Video S1 Nonmotile Tra^+^ strain (DK8601) mixed with A^+^S^−^ Tra^+^ strain (DK8615). Time-lapse microscopy was done on a Nikon E800 microscope equipped with a 20× phase contrast lens. Digital micrographs were captured on a Hamamatsu CCD camera and digitally processed with Image Pro Plus software (Media Cybernetics, Bethesda, MD, USA). Pictures were taken continuously every 30 seconds for 21 minutes. All videos were made of indicated strain mixtures (1∶1 ratio) spotted on ½ CTT agar pad supplemented with 3 mM CaCl_2_ at a calculated Klett 1000. Cells were then incubated at 33°C for 48 hrs in a humid chamber before examination. Videos were saved in mpg file format and can be viewed with Windows Media Player.
(MPG)

Video S2 Nonmotile Tra^+^ strain (DK8601) mixed with A^+^S^−^ Tra^−^ strain (DW1415). See Video S1 legend.
(MPG)

Video S3 Nonmotile Tra^−^ strain (DW1419) mixed with A^+^S^−^ Tra^+^ strain (DK8615). See Video S1 legend.
(MPG)
