# Supplementary figures and images for: Cell Contact–Dependent Outer Membrane Exchange in Myxobacteria: Genetic Determinants and Mechanism
Source: PLoS Genet. 2012 Apr 12;8(4):e1002626. doi: 10.1371/journal.pgen.1002626 (PMC3325183; doi:10.1371/journal.pgen.1002626)

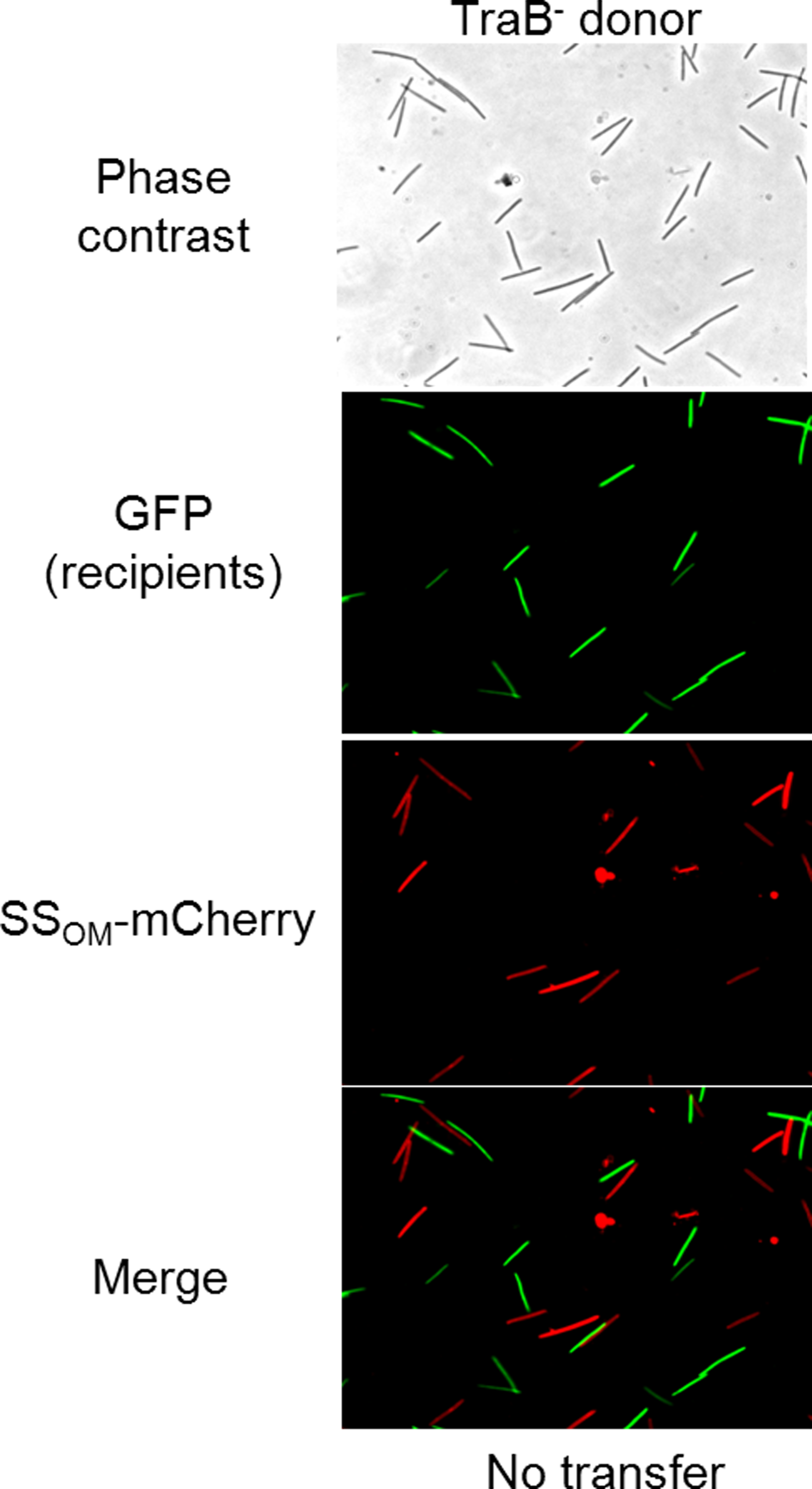

Supplement: Figure S1 — SSOM-mCherry transfer requires TraB. Strains used were DW1414 (GFP+ recipient) and DW1464 (traB::km SSOM-mCherry donor). See Figure 3 for experimental details and controls. (TIF) [file pgen.1002626.s001.tif]

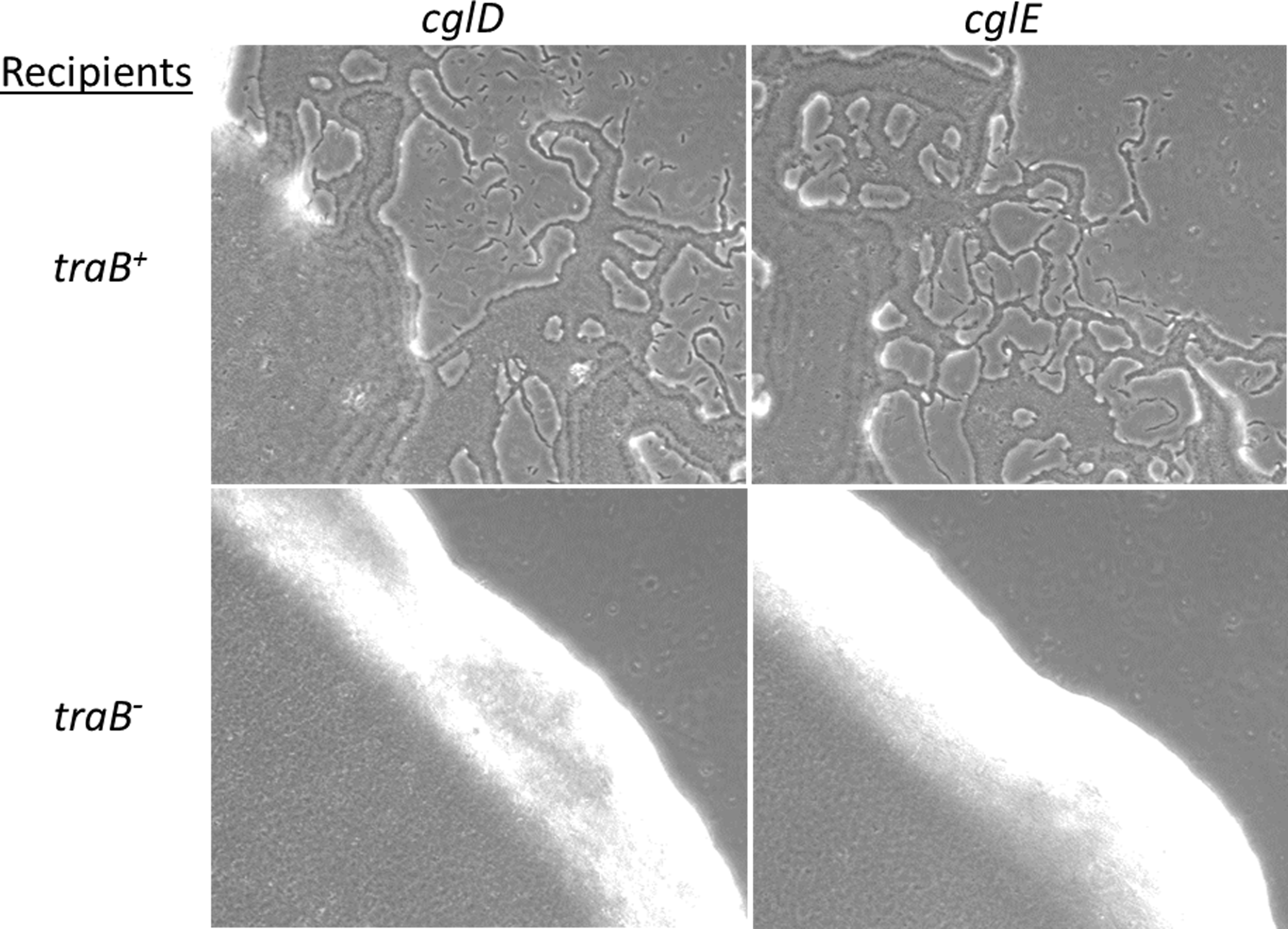

Supplement: Figure S2 — Stimulation of gliding motility depends on TraB. The nonmotile donor strain was DK8601. TraB+ recipients were DK392 (cglD1 pilQ1) and DK360 (cglE1 pilQ1). Isogenic TraB− recipients were DW1465 (DK392 traB::km) and DW1408 (DK360 traB::km). Experimental conditions were as described in Figure 1. (TIF) [file pgen.1002626.s002.tif]

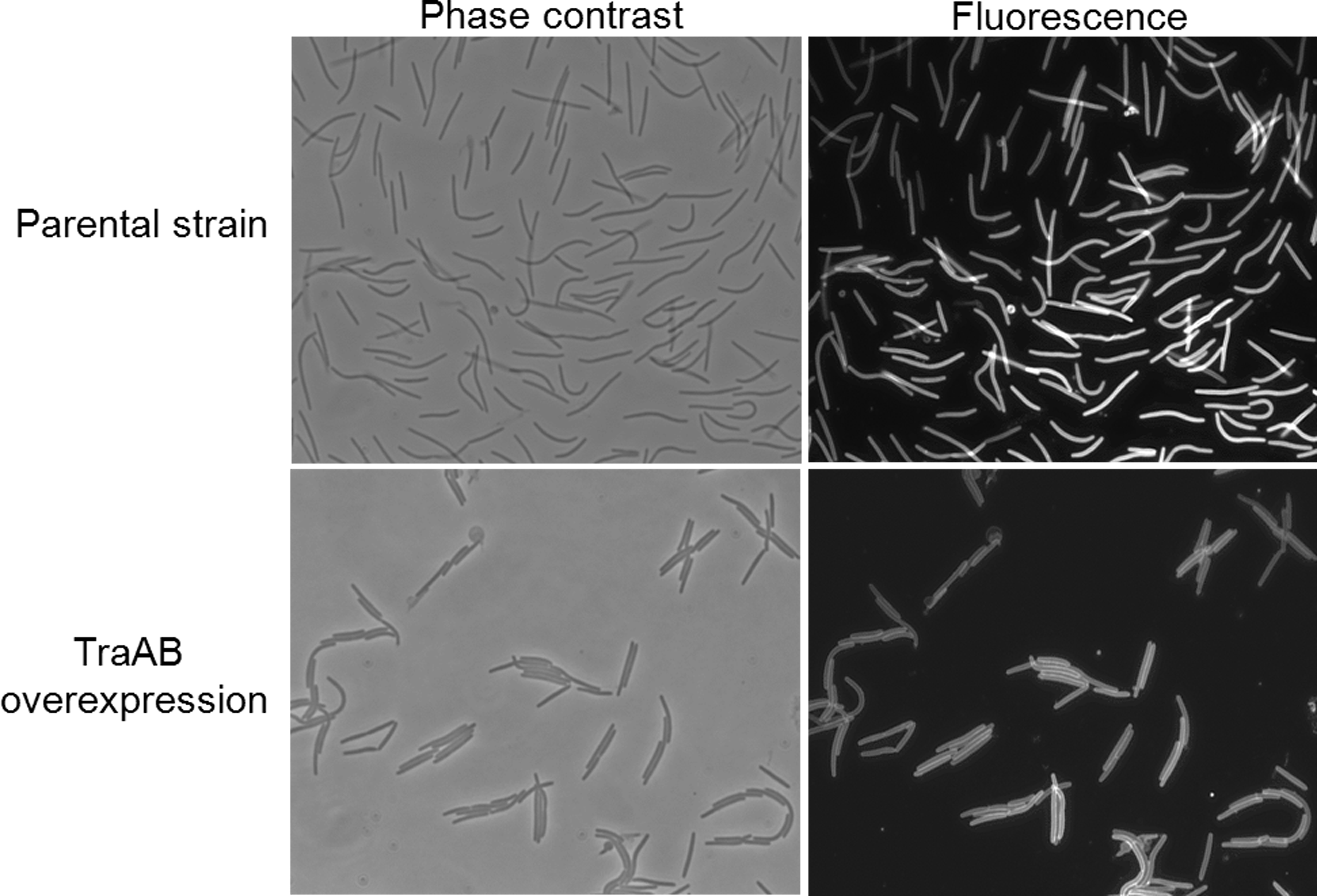

Supplement: Figure S3 — TraAB overexpression causes cells to adhere to one another. Top panels are the parental strain DW1411, which contains a ΔpilA mutation that allows disperse liquid growth and the SSOM-mCherry reporter for fluorescent OM visualization. DW1463 is an isogenic derivative that contains a second genomic copy of traAB under heterologous PpilA transcriptional control (PpilA-RBSsyn-traAB). Micrographs (100× objective) show identical phase contrast and fluorescent fields. (TIF) [file pgen.1002626.s003.tif]

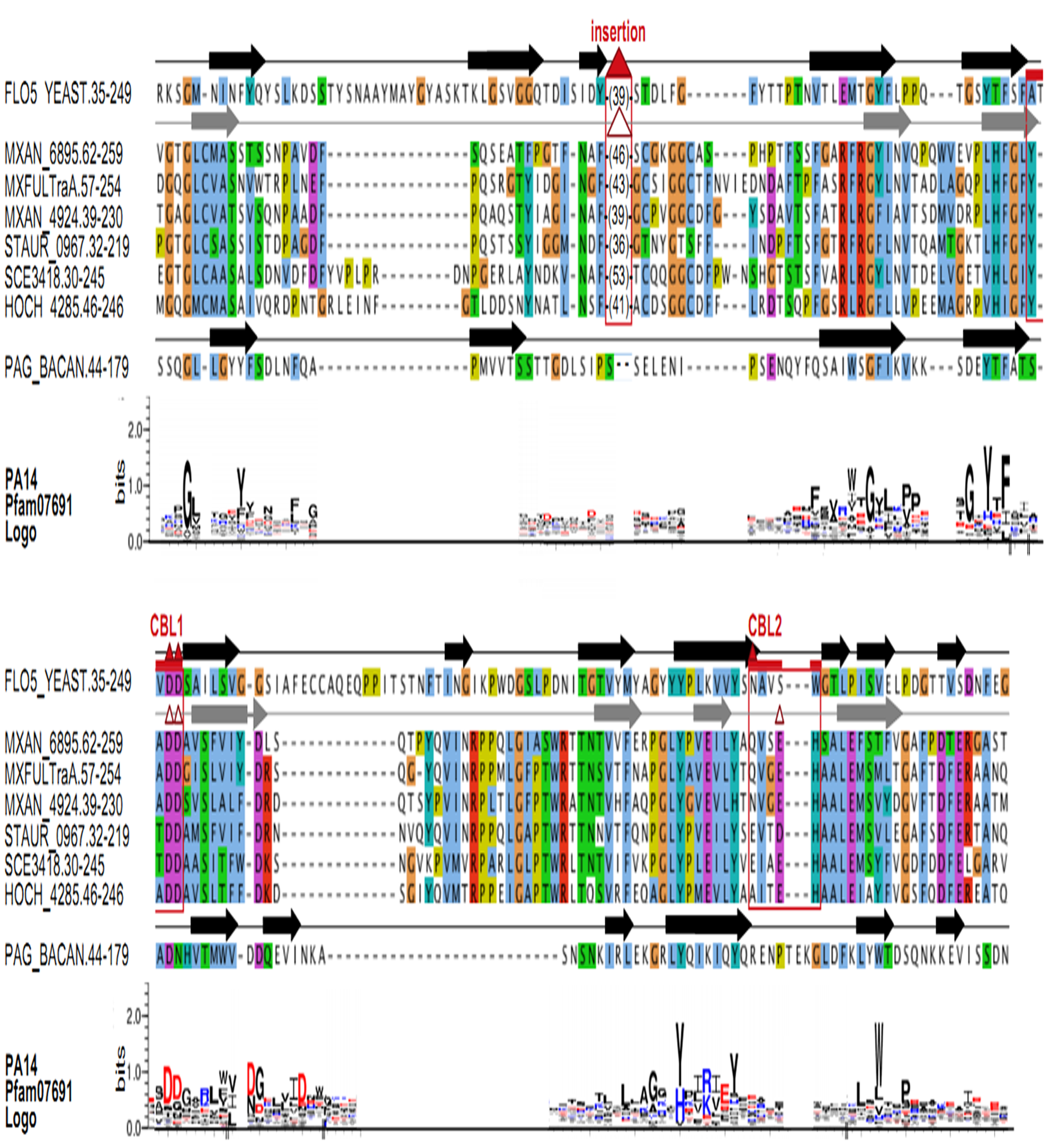

Supplement: Figure S4 — Multiple alignment extract showing Myxococcales PA14Tra domain sequences with the Pfam07691 family. The M. fulvus ORF was manually assembled from NCBI sequences. Proteins are specified by locus tags and residue numbers. The top and bottom rows shown by their Uniprot accession codes the members with high-resolution crystal structures in the PDB: FLO5_YEAST (PDB: 2XJP), PAG_BACAN (PDB: 1ACC) specifying known β-strand locations (black arrows; strands ≥3 residues). Residue coloring follows ClustalX schema to emphasize amino acid property conservation between distant homologs. Predicted β-strands (gray arrows) are from Quick2D consensus secondary structure prediction [23] on the subfamily alignment (agreement of ≥3 methods; strands ≥3 residues). No helical segments ≥5 residues are known or predicted. Similarity to previously known PA14 domains (which was used to produce the alignment, see Results) is depicted by a LOGO [62] representation of Pfam07691 after N-terminal correction based on the known structures (seed alignment, 35 sequences; this includes the structure representatives but not the new subfamily introduced by our findings). Positions with >50% gaps were excised from the LOGO (indicated by small vertical double-lines if within a block) to avoid inadequate representation by the program. The carbohydrate-binding (CB) features characterized in the flocculins [22], [24] are annotated above FLO5 (dark red triangles and lettering; CBL1 and CBL2, carbohydrate-binding loops with directly Ca2+ coordinating side chains). Red unfilled symbols markup features in the new PA14Tra subfamily that could serve a similar purpose based on homology and/or structural analogy rationales. (TIF) [file pgen.1002626.s004.tif]

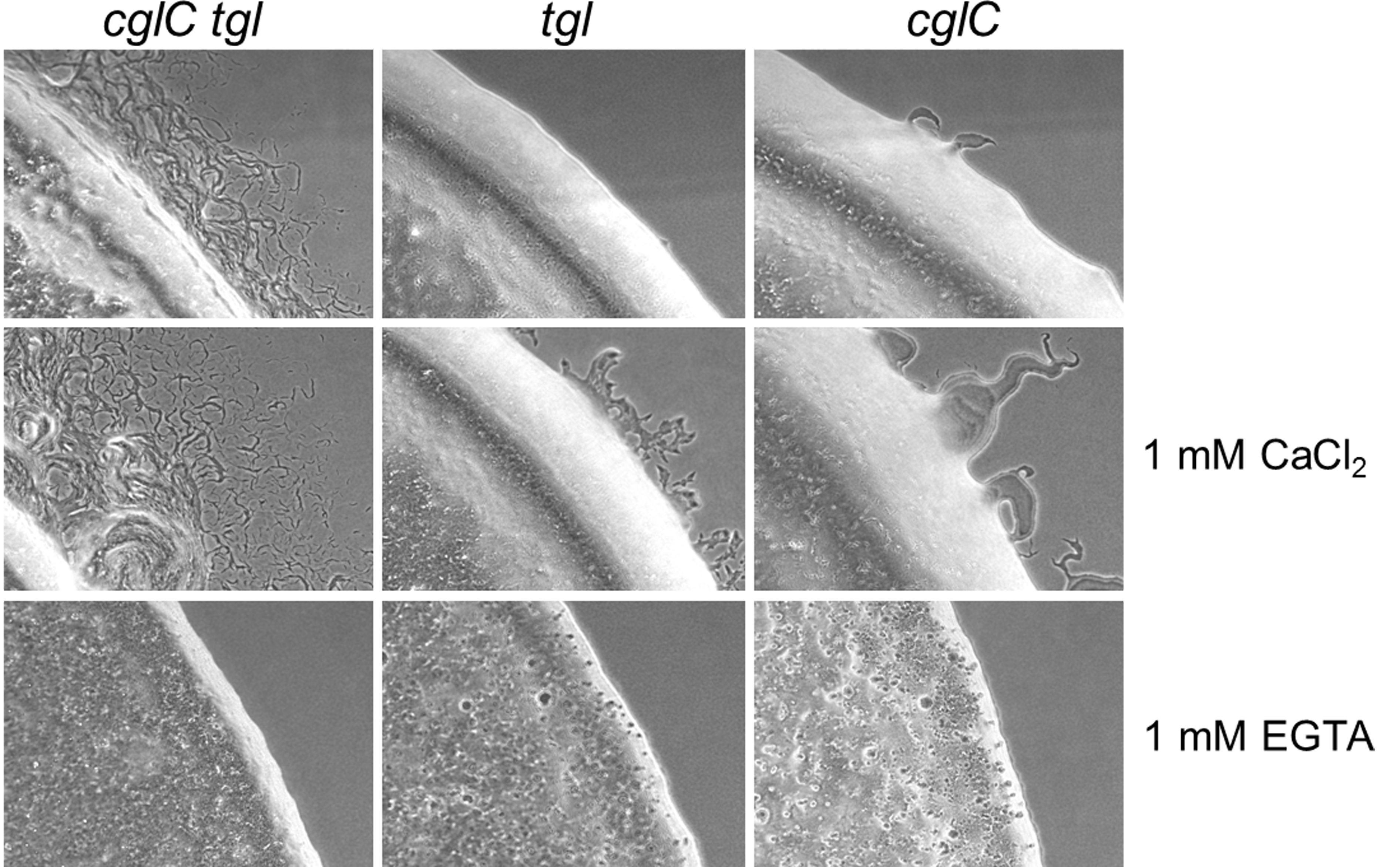

Supplement: Figure S5 — Calcium chloride enhances stimulation. A nonmotile non-stimulatable donor strain (DK8601) was mixed with the respective nonmotile but stimulatable recipient strains; DW1466 (ΔcglC Δtgl::tc), DK8602 (aglB1 Δtgl::tc) and DK1633 (cglC1 pilQ1633). Note, DW1466 can be stimulated for both A- and S-motility. Top panel assays were conducted on ½ CTT 1.0% agar, while the middle and bottom panels contained the same media with indicated supplements. See Figure 1 for details. (TIF) [file pgen.1002626.s005.tif]

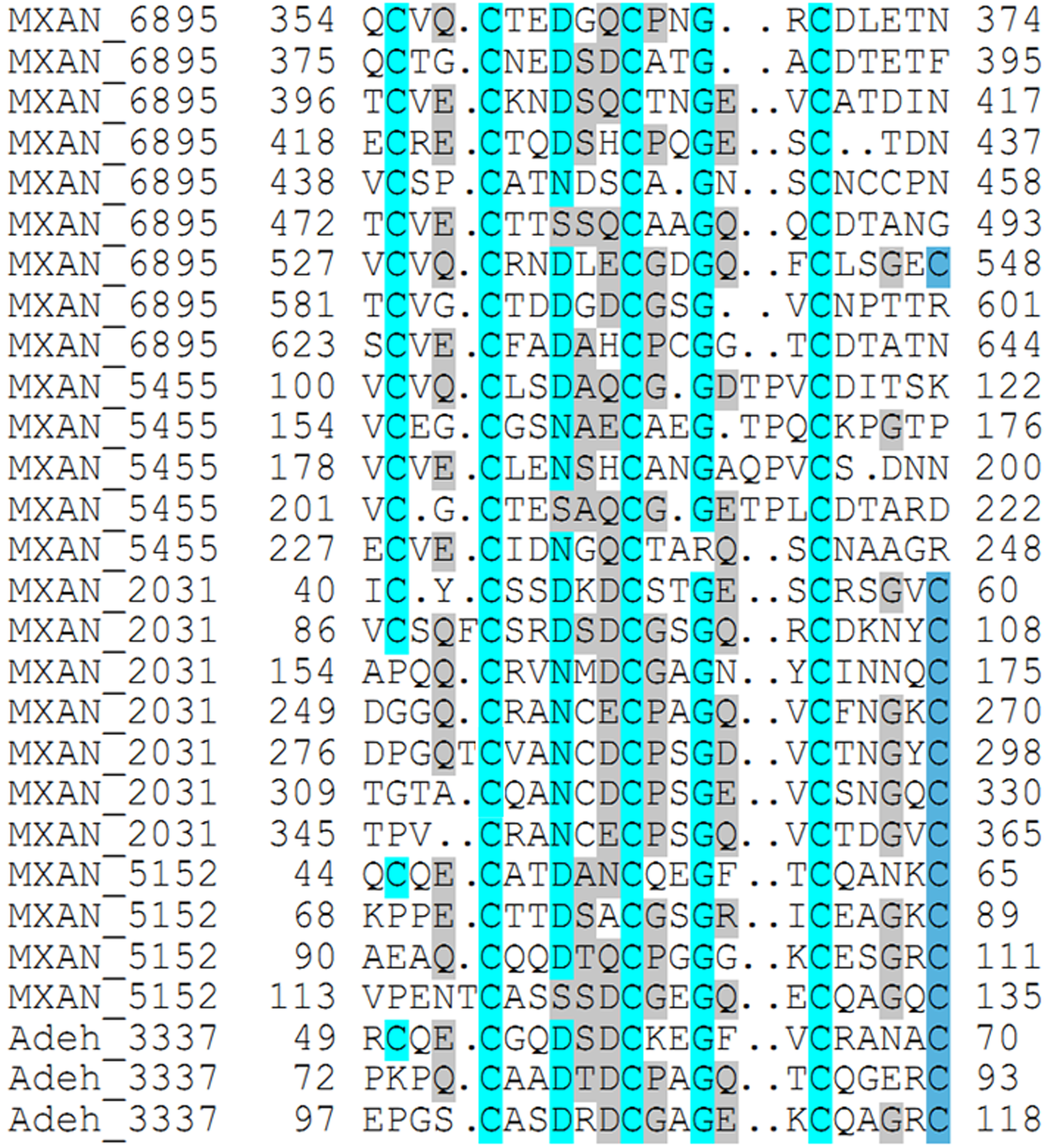

Supplement: Figure S6 — Alignment of Myxococcales Cys-rich repeats (TIGR04201). Locus tag and amino acid positions are given. Top nine sequences are from TraA (MXAN_6895), in which the first five are tandem repeats. Light blue highlights indicates highly conserved Cys residues, while dark blue highlights indicates less conserved Cys residues. Grey box shading highlights other conserved residues. (TIF) [file pgen.1002626.s006.tif]

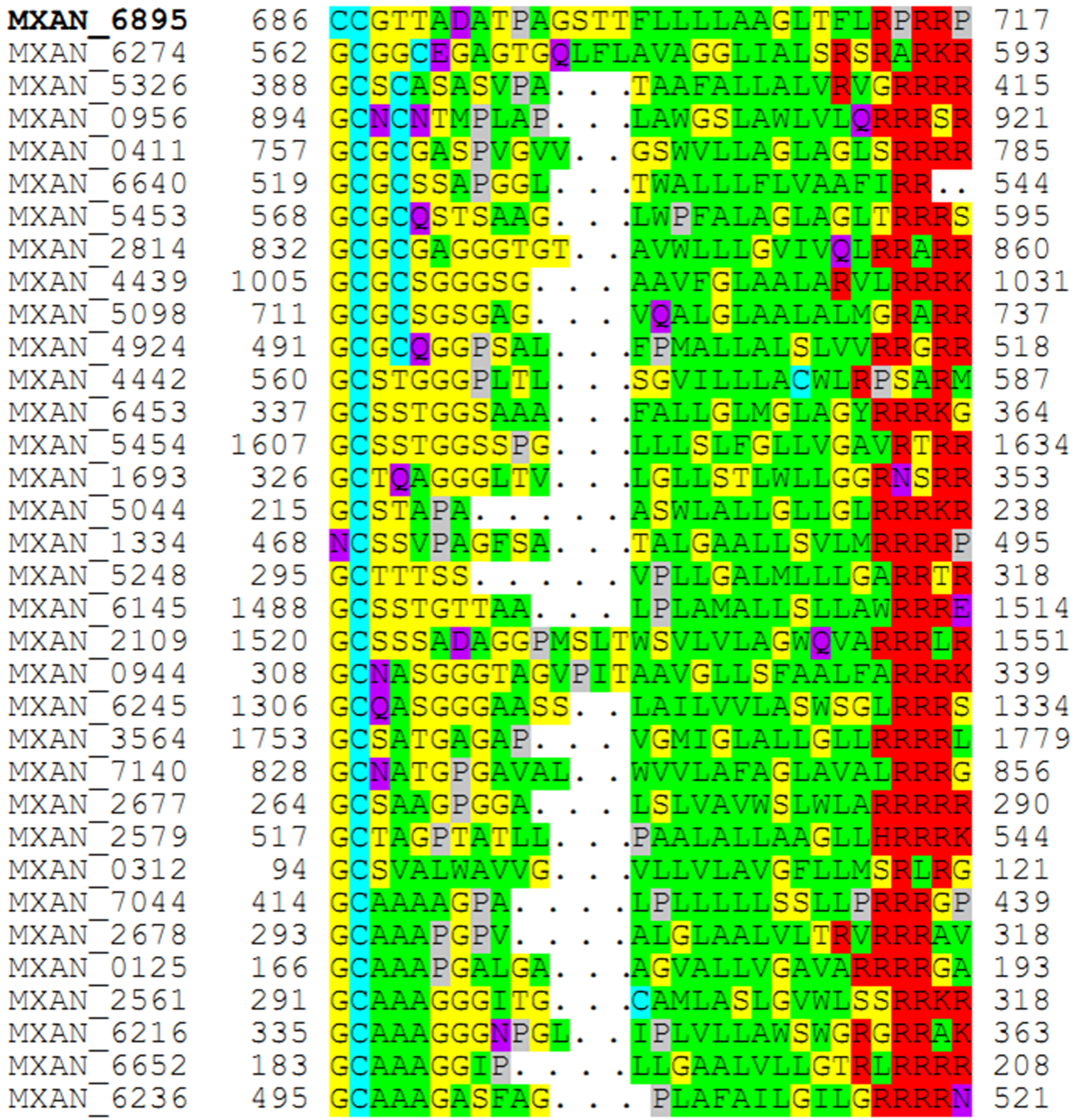

Supplement: Figure S7 — Alignment of MYXO-CTERM motif (TIGR03901) from M. xanthus DK1622 genome. Locus tags and residue positions are shown. Conserved residues highlighted. (TIF) [file pgen.1002626.s007.tif]

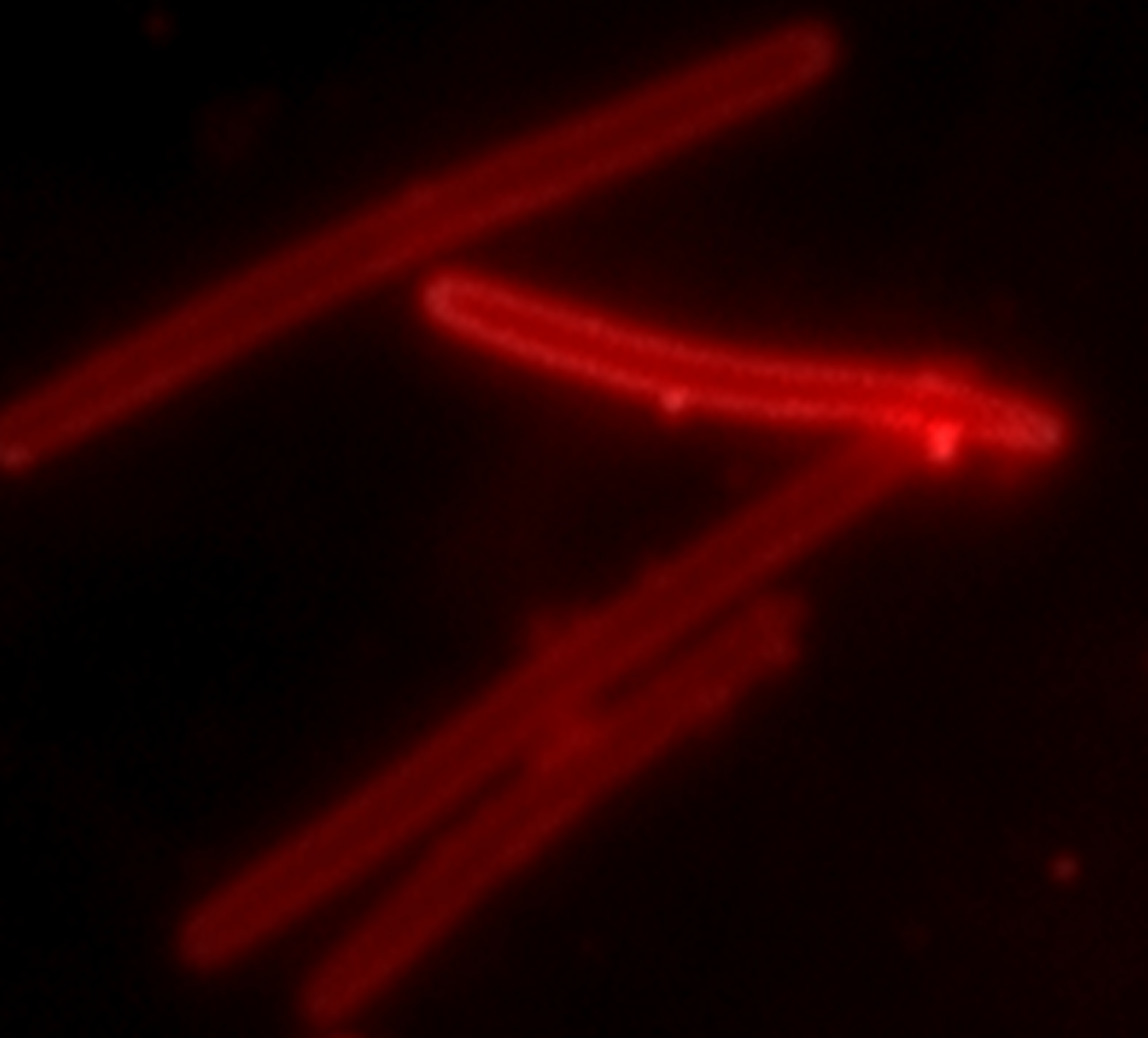

Supplement: Figure S8 — Lipophilic DiD dye stains the outer membrane. Cells observed with 100× objective. (TIF) [file pgen.1002626.s008.tif]

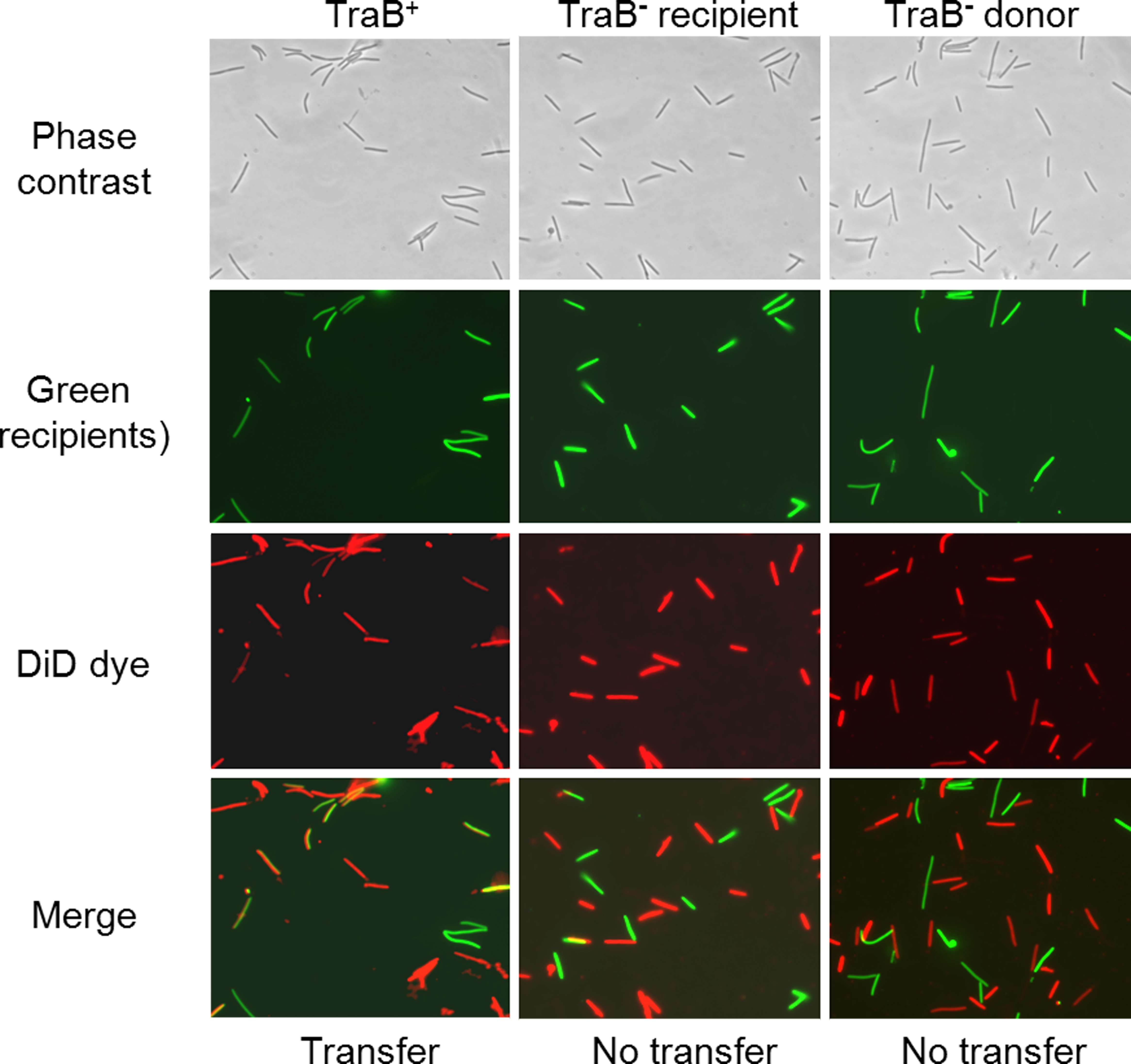

Supplement: Figure S9 — Lipophilic fluorescent dye (DiD) transfer depends on TraB in donor and recipient cells. Strains are traB+ unless indicate otherwise. The traB− strain was DW1417. See Figure 6 for details. (TIF) [file pgen.1002626.s009.tif]
