# Supplementary material for: Cell Contact–Dependent Outer Membrane Exchange in Myxobacteria: Genetic Determinants and Mechanism
Source: PLoS Genet. 2012 Apr 12;8(4):e1002626. doi: 10.1371/journal.pgen.1002626 (PMC3325183; doi:10.1371/journal.pgen.1002626)
Supplement: Table S2 — Plasmids and strains used in this study. (DOCX) [file pgen.1002626.s011.docx]

| **Plasmids** | **Relevant features** | **Source** |
| --- | --- | --- |
| pXW6 | P*_pilA_*–SS*_OM_–mCherry* cassette cloned in pKSAT, Sm^r^ | [6] |
| pDP2 | *traA* (insertion cassette) in pCR2.1, Km^r^ | This study |
| pDP3 | *traB* (insertion cassette) in pCR2.1, Km^r^ | This study |
| pDP21 | P*_pilA_*–RBS_syn_-*traAB* cloned in pSWU19, Km^r^ | This study |
| **Strains** |  |  |
| DH5α | *E. coli* cloning strain | Lab collection |
| DK1622 | A^+^S^+^, wild type *M. xanthus* | [34] |
| DK360 | A^-^S^-^, *pilQ1* *cglE1* | [11] |
| DK391 | A^-^S^-^, *pilQ1 cglD1* (leaky) | Kaiser collection |
| DK392 | A^-^S^-^, *pilQ1 cglD1* | Kaiser collection |
| DK396 | A^-^S^-^, *aglT194→NS pilR119→NS traA227P→L* | Kaiser collection |
| DK1217 | A^-^S^+^, *aglB1* | [34] |
| DK1633 | A^-^S^-^, *cglC1* *pilQ1633* | [34] |
| DK6204 | A^-^S^-^, Δ*mglBA* (markerless) | [57] |
| DK8601 | A^-^S^-^, *aglB1* Δ*pilA*::tc, Tc^r^ | [15] |
| DK8602 | A^-^S^-^, *aglB1* Δ*tgl*::tc, Tc^r^ | [15] |
| DK8606 | A^-^S^-^, *aglB1* Δ*tgl*::tc P*_pilA_*–*gfp,* Km^r^ Tc^r^ | [15] |
| DK8615 | A^+^S^-^, *∆pilQ* (markerless) | [34] |
| DK11316 | A^-^S^-^, *∆cglB* ∆*pilA* (markerless) | [53] |
| DW704 | A^-^S^-^, Δ*pilA* *cglF1,* Tc^r^ | This study |
| DW1400 | A^-^S^-^, *traA*::km, DK8602 (pDP2), Km^r^ Tc^r^ | This study |
| DW1401 | A^-^S^-^, *traA*::km, DK11316 (pDP2), Km^r^ | This study |
| DW1402 | A^-^S^-^, *traA*::km, DK1633 (pDP2), Km^r^ | This study |
| DW1403 | A^-^S^-^, *traA*::km, DK391 (pDP2), Km^r^ | This study |
| DW1404 | A^-^S^-^, *traA*::km, DK360 (pDP2), Km^r^ | This study |
| DW1405 | A^-^S^-^, *traA*::km, DW704 (pDP2), Km^r^ Tc^r^ | This study |
| DW1406 | A^-^S^-^, *traB*::km, DK8602 (pDP3), Km^r^  Tc^r^ | This study |
| DW1407 | A^-^S^-^, *traB*::km, DK391 (pDP3), Km^r^ | This study |
| DW1408 | A^-^S^-^, *traB*::km, DK360 (pDP3), Km^r^ | This study |
| DW1409 | A^-^S^-^, *traA*::km, DK6204 (pDP2), Km^r^ | This study |
| DW1410 | A^-^S^-^, *traB*::km, DK6204 (pDP3), Km^r^ | This study |
| DW1411 | A^-^S^-^, P*_pilA_*–SS*_OM_–mCherry,* DK8601 (pXW6), Sm^r^ Tc^r^ | This study |
| DW1412 | A^-^S^-^, *traA*::km, DW1411 (pDP2), Km^r^ Sm^r^ Tc^r^ | This study |
| DW1413 | A^-^S^-^, *traB*::km, DW1411 (pDP3), Km^r^ Sm^r^ Tc^r^ | This study |
| DW1414 | A^+^S^-^, P*_pilA_*–*gfp* Δ*pilA*::tc, DK8615, Km^r^ Tc^r^ | This study |
| DW1415 | A^+^S^-^, *traA*::km, DK8615 (pDP2), Km^r^ | This study |
| DW1416 | A^+^S^-^, P*_pilA_*–*gfp* Δ*pilA*::tc, DW1415, Km^r^ Tc^r^ | This study |
| DW1417 | A^+^S^-^, *traB*::km, DK8615 (pDP3), Km^r^ | This study |
| DW1418 | A^+^S^-^, *traB*::km P*_pilA_*–*gfp* Δ*pilA*::tc, DW1417, Km^r^ Tc^r^ | This study |
| DW1419 | A^-^ S^-^, *traA*::km, DK8601(pDP2), Km^r^ Tc^r^ | This study |
| DW1463 | A^-^S^-^, DW1411 (pDP21), Km^r^ Tc^r^ Sm^r^ | This study |
| DW1464 | A^-^S^-^,  *traB*::km, DW1411 (pDP3), Km^r^ Tc^r^ Sm^r^ | This study |
| DW1465 | A^-^S^-^,  *traB*::km, DK392 (pDP3), Km^r^ | This study |
| DW1466 | A^-^S^-^, Δ*tgl*::tc Δ*cglC* (markerless), Tc^r^ | This study |

Table S2. Plasmids and strains used in this study.
