# Supplementary material for: Cell Contact–Dependent Outer Membrane Exchange in Myxobacteria: Genetic Determinants and Mechanism
Source: PLoS Genet. 2012 Apr 12;8(4):e1002626. doi: 10.1371/journal.pgen.1002626 (PMC3325183; doi:10.1371/journal.pgen.1002626)
Supplement: Table S3 — Primers used in this study. (DOCX) [file pgen.1002626.s012.docx]

Table S3. Primers used in this study.

| **Primer names** | **Sequence** |
| --- | --- |
| MXAN_0736-F | CTGCTGTCCGCCGTCTAC |
| MXAN_0736-R | GTCACGTGTCACCTTCACGA |
| MXAN_1301-F | TGGCGCTGAGTTTCATCA |
| MXAN_1301-R | CAGCAGCTTCTCCAACTCCT |
| MXAN_2783-F | CTCGTCATTGGGGCGAAC |
| MXAN_2783-R | AGCACCGACTGGTAGTGGTT |
| MXAN_2884-F | CTCGACTCACACAGCGTCAT |
| MXAN_2884-R | CAGTCCTCCAGCACCAACAG |
| MXAN_3056-F | GTACGAGCTCATCCGCTTC |
| MXAN_3056-R | GAGGGCCTCCTGTTCCAG |
| MXAN_4337-F | GATGTGGTGGGCTATGTCCT |
| MXAN_4337-R | CGACCACACAGCAACTCAAA |
| MXAN_4900-F | TAGCCTCCGTCGTCTTCACT |
| MXAN_4900-R | CTTCAGCAGCTTGATCTTCG |
| MXAN_5903-F | CACCACGCTGGTGCTGTC |
| MXAN_5903-R | GTCCTCGCGCTTCTCCAC |
| MXAN_6894-F | CTGTCCTTCCGCCACCTG |
| MXAN_6894-R | CGAGCGCAACTCCAGGA |
| MXAN_6895-F | TCACTGTCTTGTCGGTGTGC |
| MXAN_6895-R | GCCGGTTGATGACCTGATAC |
| MXAN_6898-F | CTTCAACGTGCAGCTCTTCC |
| MXAN_6898-R | CGTTCTCGAAGCGGTAGTCT |
| pPilA-*Eco*RI-F | GACGACGAATTCCGTCATGTTGGACGAGGT |
| pPilA-*Xba*I-R | CAGCAGTCTAGAGCCCGCGGATGGGATTAG |
| TraA*-*RBS- *Xba*I-F | GACGACTCTAGAGGAAACCAAGAATAGAAATAGAAAGGAGAATTAGTGGGAGATATCCCTCATTG |
| TraB*-Hind*III-R | GACGACAAGCTTGGAGTTCTTCACCTCGGACTC |
